# Integrated strategies for improving lodging resistance in wheat: physiological mechanisms, plant growth regulators and agronomic management

**DOI:** 10.3389/fpls.2026.1925460

**Published:** 2026-10-12

**Authors:** Kavita Devi, Jasdev Singh Deol, Amit Kaul, Arun Kumar, Rahul Sharma

**Affiliations:** 1 Department of Agronomy, Punjab Agricultural University, Ludhiana, Punjab, India; 2 Livestock Research Station, Beechwal, Rajasthan University of Veterinary and Animal Sciences, Bikaner, Rajasthan, India

**Keywords:** agronomic management, crop physiology, culm strength, lodging resistance, plant growth regulators, root anchorage, stem biomechanics, wheat

## Abstract

Wheat lodging remains a major constraint to sustainable wheat production because it reduces grain yield and quality, increases harvesting losses and lowers production efficiency. The widespread cultivation of high-yielding cultivars, intensive nitrogen fertilization, higher planting densities, expansion of irrigated agriculture, and increasingly frequent extreme weather events have collectively increased lodging susceptibility, making effective management an important priority. Lodging is a complex phenomenon governed by interactions among plant architecture, stem mechanical properties, root anchorage, crop management practices and environmental conditions. Consequently, improving lodging resistance requires an integrated approach that combines physiological understanding with practical agronomic interventions. This review synthesizes recent advances in the structural, physiological, biochemical and agronomic mechanisms underlying lodging resistance in wheat. Particular emphasis is placed on stem biomechanics, basal internode characteristics, cell wall composition, lignin and cellulose deposition, root system architecture, and genetic factors that collectively determine plant stability. The review further examines the influence of major agronomic practices, including nitrogen management, plant population, sowing time, irrigation scheduling and cultivar selection, on lodging risk under diverse production environments. In addition, the physiological responses, field performance, practical applications and limitations of commonly used plant growth regulators, including chlormequat chloride, trinexapac-ethyl, paclobutrazol, uniconazole and ethephon, are critically evaluated in relation to their roles in improving stem strength and reducing lodging. The available evidence indicates that no single strategy provides consistent lodging control across all production systems. Instead, durable improvement in lodging resistance depends on integrating genetic improvement, balanced crop nutrition, optimized agronomic management, and judicious use of plant growth regulators according to local environmental conditions. The review also highlights emerging opportunities offered by precision agriculture, high-throughput phenotyping, advanced spray technologies, and improved understanding of stem biomechanics and root anchorage. Together, these advances provide a framework for developing sustainable, climate-resilient lodging management strategies that enhance wheat productivity, crop stability and resource-use efficiency, while also highlighting key research priorities for future investigation.

## Introduction

Increased wheat productivity can be attributed to genetic improvement and improved resource management practices. However, lodgings have become more prevalent under commercial growing conditions than in the past. High-yielding varieties produce greater amounts of biomass and heavier spikes, thus making them more likely to lodge under favourable environmental conditions Lodgings reduce grain yield and quality and, also complicate harvesting resulting in greater losses to producers through lower productivity efficiency. One especially damaging form of lodging is stem lodging. Stem lodging occurs when the culm bends or breaks near the base of the plant, particularly during critical growth stages of the crop, directly affecting canopy architecture (Mondal, 2020). Nitrogen management affects plant lodging rates. Inadequate nitrogen will reduce yield potential; conversely, excessive nitrogen will stimulate vigorous vegetative growth, thereby increasing plant height and basal internode elongation and ultimately increasing spike weight relative to stem strength. During flowering and grain formation, the stalk supports a significantly greater grain load than before flowering; therefore, high winds or heavy precipitation during this period can substantially increase lodging risk in susceptible varieties (Jin et al., 2022). The negative effects of lodging extend beyond simply bending of the stalks as once lodging occurs the canopy is no longer capable of capturing sufficient sunlight, thereby limiting the amount of photosynthesis and limiting the translocation of nutrients to the developing grains. This makes grain filling less effective, reduces kernel weight and ultimately lowers the final yield. The severity of these losses hinges on when and how much lodging occurs, with events during grain filling usually causing the most significant declines in crop productivity and increasing yield variability across the field (Jin et al., 2022; Mangin et al., 2022). Whether a wheat crop can withstand these external forces largely depends on the strength of the basal internodes. The resistance of the plant is not only based on the height, but also on how well the anatomy works to create the structural capacity to withstand mechanical forces. The way the internode walls are thickened, the arrangement of the vascular bundles and the accumulation of structural polymers in the stem all contribute to the stability of the stem under mechanical stress (Bisht et al., 2022) by enhancing the rigidity of the stem through the reinforcement of the secondary cell walls by lignin and cellulose, which increases the stem’s resistance to bending and failure in the field (Bisht et al., 2022). Lodging results from the interaction of plant structural characteristics and crop management, and therefore, agronomic practices have a direct influence on lodging incidence. Timing of planting, balanced nitrogen application, and well-designed irrigation schedules help to control the excessive vegetative development of crops and to develop thicker secondary stems that help to reduce the incidence of lodging while maintaining productivity (Zhang et al., 2023c). In addition to traditional agronomic practices used to manage lodging, the use of plant growth regulators (PGRs) has emerged as an important component of integrated lodging management. PGRs such as trinexapac-ethyl, uniconazole and chlormequat chloride have been shown to reduce the elongation of the internodes of stems, which promotes a more compact canopy architecture and is an important component of integrated lodging management (Cigel et al., 2023). The effectiveness of PGRs is not limited to the reduction of plant height, and numerous studies have shown that application at the appropriate time can also improve basal stem diameter, mechanical properties and resistance to bending in the stems of plants treated with PGRs (Cigel et al., 2023; Li et al., 2025b). Treatment responses continue to vary greatly based upon a variety of factors, including genotype, environment, timing of application, and dosage. Therefore, growth regulators should not be used as a standalone product but instead should augment balanced nutrition and water management as part of an integrated production system (Correia et al., 2025). While many studies have examined specific components of lodging, such as stem structure, biochemical composition, crop management practices, and the impact of plant growth regulators on these components, most have focused on individual aspects. However, a thorough summary of studies combining structural biology with practical crop management has not been reported (Bisht et al., 2022). This review synthesizes the various elements of these complementary research areas, providing a critical assessment of how PGRs enhance lodging resistance through changes in stem morphology, mechanical characteristics and hormonal control. This highlights their importance as part of the total nutrient and irrigation management system for sustainable wheat production (Li et al., 2025a).

## Literature search and selection criteria

A structured literature search was conducted to identify research relevant to lodging resistance in wheat, with emphasis on stem biomechanics, root anchorage, cell-wall composition, plant growth regulators, agronomic management, genetic improvement and emerging phenotyping technologies. Relevant literature was identified from major scientific databases and scholarly search platforms using combinations of keywords including “wheat lodging”, “stem lodging”, “root lodging”, “lodging resistance”, “stem strength”, “root anchorage”, “plant growth regulators”, “nitrogen management”, “agronomic management”, “QTL”, “genomic selection”, “candidate genes”, and “genome editing”. Priority was given to peer-reviewed studies directly examining wheat, while evidence from other cereal crops was considered only where it provided relevant mechanistic or comparative information and was clearly distinguished from wheat-specific evidence. Studies were selected based on their relevance to the review objectives, experimental evidence, methodological clarity and contribution to understanding the mechanisms or management of lodging resistance. The selected literature was organized thematically and synthesized across physiological, structural, biochemical, genetic and agronomic dimensions to identify consistent responses, sources of variation and knowledge gaps.

## Types and mechanics of lodging in wheat

### Forms of lodging: stem bending vs. root lodging

In wheat, lodging may occur either through failure of the stem itself or through instability of the root system under external mechanical pressure. Stem lodging is commonly associated with bending or breakage of culms when the aerial portion of the crop can no longer withstand forces generated by wind, rainfall or spike weight (Shah et al., 2019). By contrast, root lodging develops when anchorage between the roots and surrounding soil becomes inadequate, causing the whole plant to lean or collapse, particularly in fields experiencing excessive soil moisture or weakened soil structure (Rabieyan et al., 2024a). These two forms of lodging differ not only in their physical appearance but also in the underlying factors responsible for crop failure. Reduced stem wall thickness and narrower culm diameter generally increase susceptibility to stem lodging, whereas poor root spread and unstable root-soil contact contribute more strongly to root lodging (Rabieyan et al., 2024a). Understanding these differences is important when developing lodging-management strategies because practices that are effective against stem lodging do not always prevent root lodging. Practices designed to strengthen stem tissues and improve culm strength are more effective for reducing stem failure, whereas improving root traits and maintaining favourable soil conditions are important for reducing root lodging (Rabieyan et al., 2024a). The relationship between root lodging in wheat and the anchorage provided by crown and nodal roots under adverse weather conditions is strong (Klepper et al., 1984; Zhang et al., 2006). Wheat roots can reach depths of up to 2 m under favourable soil conditions (Klepper et al., 1984). Although roots can reach greater depths, Zhang et al. (2006) and Wang et al. (2014) reported that approximately 87.4 per cent of the root system is concentrated within the upper 0–40 cm of soil. Such shallow root concentration weakens root-soil anchorage under saturated soil conditions and increases susceptibility to root lodging during heavy rainfall and strong winds (Wang et al., 2014). These observations indicate that lodging resistance in wheat depends not only on culm mechanical strength but also on effective belowground anchorage (Klepper et al., 1984; Wang et al., 2014). In addition to stem mechanical properties, root architectural traits also contribute substantially to lodging resistance in wheat by influencing anchorage stability and resistance to overturning forces (Zhang et al., 2006; Wang et al., 2014). Important root related characteristics associated with lodging resistance in wheat are summarized in **Table 1**.

**Table 1:** Root architectural traits associated with lodging resistance in wheat.

Root trait	Quantitative observation	Contribution to lodging resistance	Reference
Root depth distribution	~87.4 per cent roots concentrated within upper 0–40 cm soil layer	Shallow rooting may weaken anchorage under saturated soils	(Zhang et al., 2006); Wang et al., 2014)
Maximum rooting depth	Roots may extend up to ~2 m under favourable conditions	Deeper rooting improves plant anchorage and stability	(Klepper et al., 1984)
Root distribution pattern	92.2-96.3 per cent roots distributed within upper 0–80 cm soil profile	Surface root concentration may increase root lodging susceptibility	(Li et al., 2018)
Root weight density	Maximum value of 2.7 × 10–^4^ g cm^-3^ observed under moderate soil moisture conditions	Greater root density may improve belowground support	(Man et al., 2016)

**Figure 1** presents the influence of external mechanical forces, including rainfall, wind pressure, plant stature and ear weight, on the development of basal-internode and root lodging in wheat. The major categories of lodging together with their distinguishing characteristics are summarized in **Table 2**.

**Figure 1:**
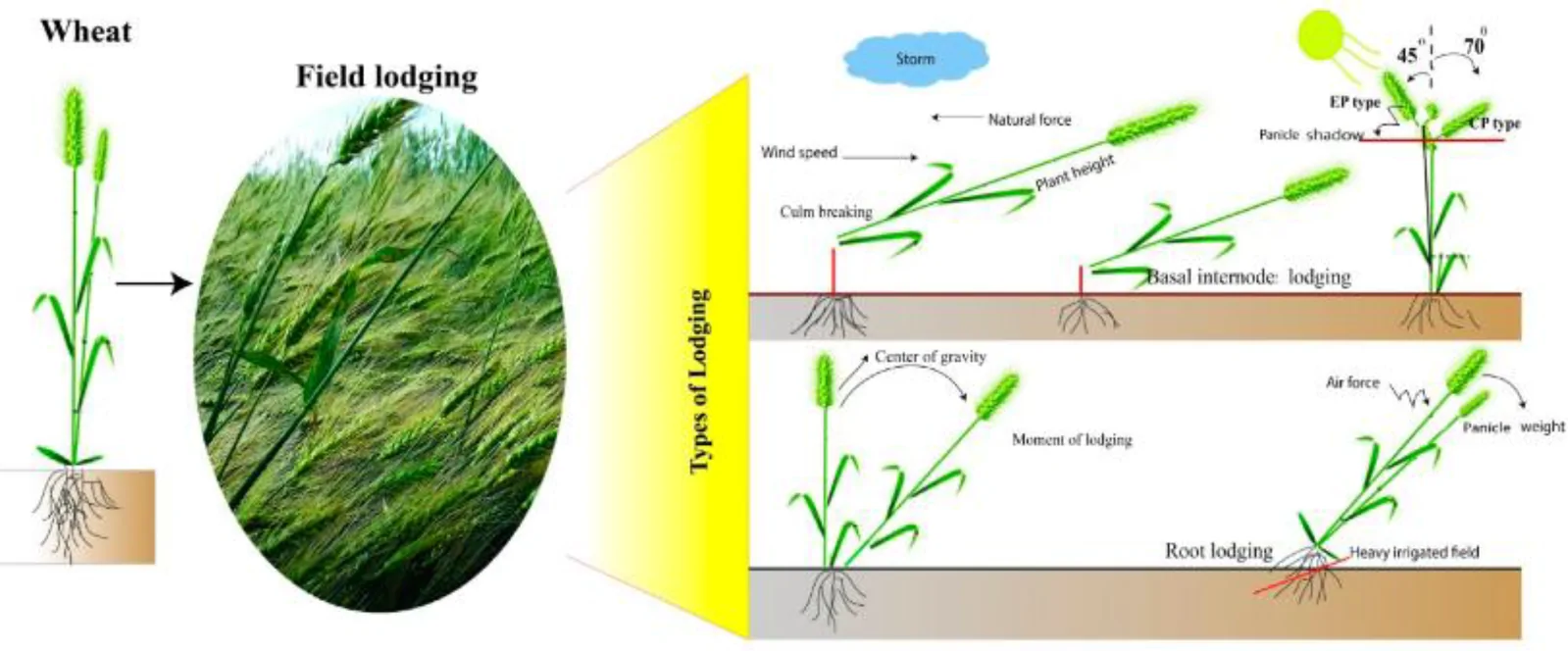
Types of lodging and their mechanical determinants in wheat. Adapted from (Shah et al., 2019).

**Table 2:** Different categories and key characteristics of lodging in wheat.

Lodging type	Definition/main feature	Primary causes	Typical field symptoms	References
Stem lodging	Bending or buckling of the lower stem internodes while roots remain firmly anchored in the soil.	Excessive plant height, weak basal internodes, high nitrogen, strong wind and rain during stem elongation or grain filling.	Stems bent or broken near the base; plants often lie close to the ground but roots stay in place; common in tall, lush crops.	(Berry and Spink, 2012; Wu et al., 2016; Niu et al., 2022b)
Root lodging	Tilting of relatively intact stems due to failure of root anchorage at or near the crown.	Shallow or weak root systems, waterlogging, loose or saturated soil, strong winds after heavy rain.	Plants lean from the base with intact stems; whole root plate rotated in the soil; lodging often occurs in patches after storms.	(Berry and Spink, 2012; Wu et al., 2016; Mondal, 2020)
Mixed lodging	Combination of stem and root lodging occurring together in the same crop.	Very tall, dense crops exposed to wind/rain where both stem strength and root anchorage are inadequate.	Irregular lodging pattern: some stems broken at the base, others leaning with uprooted crowns; often seen under high-input management.	(Shah et al., 2019; Niu et al., 2022b)
Brackling/head lodging (mainly in some cereals)	Bending or kinking of the upper stem or neck region near the spike, causing ear heads to fall over.	Weak peduncle or neck region, heavy spikes, disease or physical damage in upper internodes.	Spikes bent over or touching the ground while lower stem and root remain relatively upright; more common in barley/oats but can occur in wheat.	(AHDB, 2023; Niu et al., 2022b)

### External driving forces and lodging mechanics

The development of lodging in wheat is closely associated with an imbalance between externally imposed mechanical forces and the inherent structural strength of the plant. Under field conditions, wheat stems are continuously exposed to environmental stresses, among which wind and rainfall are considered the most damaging (Shah et al., 2019). The lateral pressure exerted by strong winds on the canopy and the resulting bending forces on the stem can be amplified by the additional weight of rainwater retained on the canopy and the increased gravitational load on the basal portions of the stem. These forces can exceed the structural capacity of the stem or anchorage capacity of the root system, resulting in permanent stem bending or root lodging (Shah et al., 2019). The probability of structural failure occurring during booting and heading stages is high because of the rapid elongation of the stem during these periods, leading to mechanically weaker and more vulnerable internodes than at other times. Stem resistance to bending during the booting and heading stages depends largely on culm geometry, tissue rigidity, and the ability of the basal internodes to withstand weather-related bending forces (Shah et al., 2019).

### Key morphological traits influencing lodging

Morphological characteristics such as plant height, stem diameter and culm wall thickness are widely recognized as primary determinants of lodging resistance, with taller plants and narrow stems generally exhibiting increased susceptibility to lodging (Shah et al., 2019).

### Cell-wall biochemistry and mechanical resistance

In addition to the geometry of the stem, the biochemical makeup of the stem tissue also influences the lodging resistance of the wheat plant. The rigidity of the cell wall is determined to a great extent by its lignin and cellulose content. Cellulose provides tensile strength, while lignin adds more stiffness and structural support to a stem to help it withstand bending forces (Mengistie and McDonald, 2024). An accumulation of both lignin and cellulose has been associated with greater stem strength and lodging resistance in various cereal crops. Several studies have found no consistent relationship between hemicellulose content and lodging (Mengistie and McDonald, 2024). Factors associated with lodging resistance and related plant traits are presented in **Figure 2** and **Table 3**.

**Figure 2:**
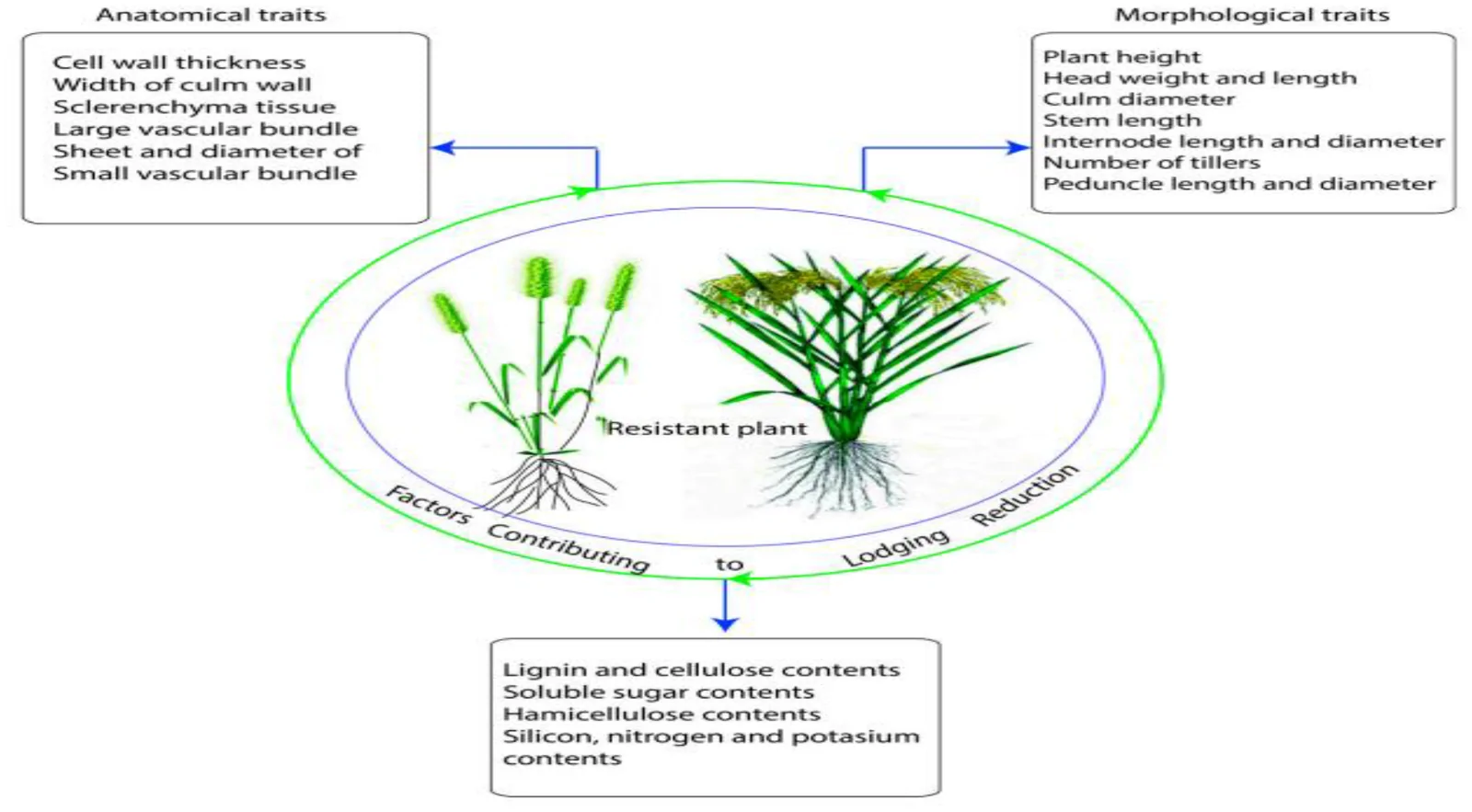
Factors associated with the lodging resistance. Adapted from (Shah et al., 2019).

**Table 3:** Plant characteristics correlated with lodging resistance (Shah et al., 2019).

Category	Trait	Crop	Behaviour/relationship with lodging
1. Morphological features	Plant height	Wheat and rice	Positively correlated with lodging
	Primary internode length	Wheat and rice	Negatively correlated with lodging resistance
	Culm diameter	Wheat and rice	Strong positive association with lodging resistance
	Culm length	Wheat and rice	Positively correlated with lodging
	Culm diameter, panicle weight, and panicle length	Rice	Positively correlated with lodging resistance
2. Anatomical aspects	Number of vascular bundles	Rice	Strong positive association with lodging resistance
	Width of mechanical tissue layer	Wheat	Strong positive association with lodging resistance
3. Biochemical features	Lignin and cellulose contents	Wheat and rice	Strong positive correlation with culm strength, secondary cell-wall strength, and lodging resistance

## Physiological and biochemical pathways underlying lodging resistance

Lodging resistance in wheat is affected by numerous physiological and biochemical processes, in addition to visible structural characteristics. Gibberellins are the principal plant hormones associated with stem elongation via promotion of both cell division and cell expansion within developing internodes. Higher levels of gibberellin activity generally result in taller plants with longer stems; these conditions increase the centre of gravity and thereby increase the probability of lodging. On the other hand, inhibiting gibberellin biosynthesis limits excessive stem elongation and produces culms with greater strength, thus establishing the physiological basis for the majority of lodging-control growth regulators used on cereals (Hedden and Sponsel, 2015). Ethylene hormones generally inhibit longitudinal stem growth by stimulating additional radial stem growth; therefore, moderate levels of ethylene may contribute to the development of thicker culm walls and better mechanical support. In contrast, excessive ethylene production due to environmental stresses can accelerate senescence and shorten the length of time leaves are able to produce photosynthates. The ratio of gibberellin to ethylene activity is significant in determining whether assimilates are directed toward stem elongation or toward reinforcement of supportive tissue (Yang and Hoffman, 1984). Cytokinins also indirectly influence lodging by affecting the number of tillers and the size of canopy developed and, therefore, the distribution of assimilates within plants. These indirect effects ultimately impact the relationship between the load on the ear and the capacity of the stem to support the load (Jameson and Song, 2016). Biochemical strengthening of the culm is mainly associated with the accumulation of lignin, cellulose and hemicellulose within stem tissues. Deposition of lignin in secondary cell walls, particularly in basal internodes, improves rigidity and resistance against bending forces. Several studies in cereals have reported positive associations between increased lignin or cellulose concentration and enhanced stem breaking strength together with lower lodging incidence (Bisht et al., 2022). The synthesis of lignin is regulated through enzymes of the phenylpropanoid pathway, including phenylalanine ammonia-lyase, cinnamyl-alcohol dehydrogenase and various peroxidases. Their activity can be modified by both nitrogen availability and plant growth regulator application.

The physiological basis of lodging resistance is also closely associated with the distribution of carbohydrates within the plant. The accumulation of non-structural carbohydrates within stem tissues contributes to stem thickening and also provides carbohydrate reserves for grain filling after anthesis. Rapid assimilate allocation to developing spikes, when not accompanied by sufficient strengthening of the basal internodes, can considerably increase the mechanical load on the stem and make plants more susceptible to lodging (Zuber et al., 1999). Lodging resistance in wheat is influenced by hormonal regulation of stem biochemical composition and by the allocation of carbon among processes involved in plant growth and development.

## Structural and biochemical basis of lodging resistance in wheat

### Anatomical reinforcement of basal stems

Frequent improvements in lodging resistance of wheat have resulted from stronger culm architecture due to thicker stem walls, wider culm diameter, and better developed supporting tissues of genotypes (Feng et al., 2023; Zheng et al., 2023). The stem anatomical characteristics help stems tolerate bending pressure and reduce buckling under extreme weather conditions. These structural improvements also include increased accumulation of structural compounds within the basal internodes, contributing to greater stem rigidity and lodging resistance (Li et al., 2022c; Feng et al., 2023).

### Cell wall composition: lignin, cellulose and hemicellulose

There is a strong relationship between lodging resistance and the accumulation of structural polymers in wheat stem tissues. The primary cell-wall components-lignin, cellulose, and hemicellulose-contribute substantially to the ability of wheat stems to resist bending by increasing rigidity and providing mechanical stability (Li et al., 2022c; Zhang et al., 2023c). For example, lignin provides increased compressive strength due to its interaction with the cellulose and hemicellulose polysaccharides in the cell wall and increases tensile strength due to its interaction with cellulose microfibrils in the stem. In general, wheat plants with higher concentrations of these compounds have stronger culms than those with lower concentrations (Mengistie and McDonald, 2024; Li et al., 2025a) and similar relationships have been reported in other cereal crops.

### Structural carbohydrate enhancement through management

Crop management practices can substantially alter the internal structure of wheat stems and consequently influence lodging behaviour. Studies have reported that delayed sowing or increased plant density can alter the accumulation of lignin and cellulose in wheat stem tissues (Li et al., 2025a; Zhang et al., 2023c). These structural changes can strengthen wheat stems and improve lodging resistance by reinforcing internal stem tissues. These management practices may also increase the availability of substrates for cell-wall formation and strengthen structural connections within the stem. Field studies have reported increases in wheat stem breaking strength and structural carbohydrate content under these conditions. Many scientists believe that lodging resistance is modified through field management actions, even though genetic improvement is also important (Li et al., 2025b; Zheng et al., 2023).

### Morphological integration and resistance phenotypes

Lodging resistance in wheat depends on the integration of stem anatomical and structural traits because no single characteristic is sufficient to provide adequate protection. Wheat genotypes with wider stem walls, denser tissues, and greater accumulation of structural compounds exhibit greater resistance to bending forces and lodging (Feng et al., 2023; Li et al., 2022c). It has been observed that these physical features assist in keeping stem shape from changing when weight is applied. These structural traits contribute to plant stability throughout crop development. Studies from high-yielding production environments indicate that these mechanical advantages are associated with improved grain production and greater plant stability. Lodging resistance in wheat reflects overall plant structural strength, as plant height alone does not determine lodging susceptibility (Zheng et al., 2023; Mengistie and McDonald, 2024).

### Biochemical regulation of reinforcement pathways

Surrounding natural situations together with developmental and physiological signals change how structural compounds are produced within wheat stems. It has been observed that pathways involved in lignin, cellulose, and hemicellulose synthesis change in response to fluctuations in nutrient availability, hormonal balance, and stress factors, leading to changes in stem structural properties (Li et al., 2022c; Zhang et al., 2023c). Molecular studies have shown that higher activity in genes for cellulose synthase and the phenylpropanoid pathway makes stem tissues more powerful and helps lodging resistance. Stem reinforcement can be achieved through physiological regulation instead of just breeding interventions (Mengistie and McDonald, 2024; Feng et al., 2023). Biochemical strengthening of the culm represents a significant part of lodging resistance, particularly when lignin build-up increases within sclerenchymatous tissues. Stem stiffness and bending resistance are improved by higher lignification, which decreases the chance that plants will fall over during outdoor farm environments (Zheng et al., 2017). Certain growth-regulating compounds, such as paclobutrazol and uniconazole, have been reported to enhance the activity of lignin-biosynthetic enzymes, including PAL, CAD, POD, and 4CL. These substances produce thicker stem walls and better stem breaking strength (Ma et al., 2021; Li et al., 2022b).

## Genetic and varietal approaches to lodging resistance in wheat

The genetic improvement of long-lasting lodging resistance is the best way to achieve long-term lodging resistance improvements. The agronomic practices used will reduce lodging only under certain circumstances, but will not address the inherent weakness of the cultivar being used. Management strategies will not replace the structural stability created through genetics. The ability of wheat to resist lodging is a result of several interacting elements or traits that include stem strength, plant architecture, and root anchorage. There is considerable variation in these traits among cultivated wheat varieties and wheat landraces. This variability provides breeders with a broad genetic pool for developing lodging-resistant cultivars (Rabieyan et al., 2024b). The introduction of modern genomic technologies adds even more value to this genetic diversity. Researchers have used high throughput genotyping and whole genome association studies, along with genomic predictions to identify many genomic regions associated with lodging-related traits in various wheat populations. As indicated by Rabieyan et al. (2024b), molecular technologies provide new opportunities to integrate lodging resistance into breeding programs through marker-assisted and genomic selection. This provides an improvement from previous traditional breeding methods which were based primarily on field evaluations where lodging occurs naturally. Varietal differences due to environmental variability often reduced the degree of effectiveness and consistency of previous evaluations.

Lodging tolerance can be improved through breeding for favourable stem structural traits. Wheat cultivars with a broader stem base, thicker walls, stronger lower internodes, better developed root systems, and a moderate (medium) height have greater resistance to lodging than tall varieties with weaker stem structures. Since many of the traits that affect lodging tolerance can be passed on through genetic inheritance, they can be used in early breeding selections. Selecting for lodging resistant candidates at early breeding stages will allow more potential lodging resistant cultivars to be found before conducting many yield trials in different environments (Rabieyan et al., 2024b). Anatomical work has provided additional information regarding the structural characteristics of the stem that contribute to the resistance to lodging. The number of vascular bundles and their arrangement near the bottom of the internode are correlated to the stiffness of the stems; therefore, it appears that the internal architecture of the stem contributes to the mechanical strength of stems through structural features (Niu et al., 2022a). Both anatomical traits can be assessed through traditional phenotypic evaluation and molecular breeding approaches, thereby expanding the options available for selection. Lodging resistance is also a quantitative trait influenced by multiple genomic regions rather than a single major gene. Quantitative trait loci have been identified on chromosomes 2D, 3A, 3B, 4B, 5A, 6B, and 7A for characteristics associated with stem diameter, culm wall thickness and vascular bundle traits (Niu et al., 2022a). This indicates that lasting genetic improvement in lodging resistance will require the integration of favourable alleles from multiple loci rather than selection for individual genes alone. Thus, it may be concluded that making improvements in lodging resistance will involve obtaining favourable alleles from various loci within the genome. Marker-assisted selection is a useful method for combining beneficial genomic areas linked to stronger stems while also ensuring high yield potential. In addition to traditional QTL-based methods, researchers are increasingly looking at genomic prediction models for traits related to lodging. These models can assess the effects of multiple genome-wide markers at once. They can capture the influence of minor genetic factors that together affect lodging behaviour, which may enhance selection efficiency in genetically diverse breeding populations (Rabieyan et al., 2024b). These models are particularly useful for predicting complex traits that are affected by strong interactions between genotype and environment. Recent studies have also aimed to identify stable meta-QTL and candidate genes linked to lodging resistance by combining multiple QTL datasets. This method increases the dependability of the genomic regions detected across different environments and genetic backgrounds. Genes identified within these consensus regions are frequently associated with vascular development and cell-wall-related processes, including lignin and cellulose metabolism. Recent work has provided experimental expression-level validation of candidate genes associated with lodging tolerance: Duhan et al. (2024) prioritized TraesCS2D02G256600 (TaEREB114-2D), TraesCS7B02G340100 (TaIRX14-7B), and TraesCS1B02G347600 (TaGA2ox4-1B) and validated their expression using qRT-PCR, with all three genes showing higher relative expression in the second basal internode of the lodging-tolerant cultivar than in the lodging-susceptible cultivar. In addition, Li et al. (2024) identified 14 significant SNPs and 13 QTLs for cellulose crystallinity in wheat stems and prioritized TraesCS4B03G0029800 and TraesCS5B03G1085500 as candidate genes, providing further genetic evidence linking cell-wall properties with stem strength and lodging resistance. Genome-editing approaches, particularly CRISPR/Cas9, provide an additional tool for functional validation of genes controlling plant architecture and lodging-related traits. In wheat, CRISPR/Cas9-mediated knockout of TaDHL-7B, a newly identified reduced-height gene, significantly reduced plant height without a yield penalty, demonstrating the potential of targeted genome editing to modify plant stature and provide genetic resources for lodging-resistant breeding (Guo et al. (2022). Recent advances in wheat genome editing, including multiplex CRISPR/Cas9, base editing and prime editing, further provide opportunities for precisely modifying multiple homoeologous gene copies and validating candidate genes identified through QTL and genomic studies (Li et al., 2021).

Comparisons between historical and modern wheat varieties show the progress made through ongoing breeding efforts. Newly developed varieties usually have thicker stems, stronger walls, and lower lodging indices compared to older ones. The long-term evolution of wheat breeding has seen an increase in lodging resistance, with modern wheat varieties usually featuring structural stability under intensive production systems due to decades of direct and indirect selection for desirable stems (Wang et al., 2023). Thus, lodging resistance should be viewed as a distinct breeding objective rather than solely as a by-product of selection for higher grain yield. Further improvement in lodging resistance also depends on advances in tools for its evaluation and selection. Visual assessment of lodging-related traits has gradually been supplemented with image-based phenotyping platforms, which allow for quicker and more consistent assessments of larger breeding populations compared to traditional evaluation methods in different agroecological zones. Likewise, using machine learning has improved genotype identification by extracting latent patterns from high-throughput phenotypic data that may not be otherwise apparent when evaluating manually. Advances in molecular breeding, genomic prediction, and marker-assisted selection have also enabled breeders to identify favourable alleles for stem strength and other structural traits earlier in breeding programs, reducing reliance on later field-based lodging evaluations. Genetic studies have shown that lodging-related traits differ substantially in their heritability, with stem-strength traits showing relatively high heritability of 0.64-0.95, compared with 0.21-0.33 for root-anchorage traits, indicating greater environmental influence on root anchorage (Piñera-Chavez et al., 2021). In another doubled-haploid population, 15 QTL were identified for stem-strength traits including vascular-bundle number, stem diameter and stem-wall thickness, and the superior allele of a major vascular-bundle QTL was independent of plant height, indicating the possibility of improving stem strength without increasing plant stature (Niu et al., 2022a). Together, physiological ideotype breeding and genomic tools represent a system of integrating traits that need to be incorporated into future wheat cultivars for sustainable lodging resistance.

## Plant growth regulators and their mechanisms in improving lodging resistance in wheat

Most growth regulators used for wheat function primarily by altering gibberellin production (Hedden and Thomas, 2012; Peng et al., 2025). When gibberellin production decreases, internode elongation is reduced, resulting in shorter and more mechanically stable stems. These structural changes allow the base of the stem to experience less mechanical force from the canopy of the crop above. This approach has become increasingly relevant to contemporary wheat production systems with a high risk of lodging. Agricultural methods such as excessive nitrogen fertilization and frequent irrigation cause rapid biomass growth and put crops at risk for lodging caused by excessive stem elongation. Therefore, the use of growth regulators in these environments can modify canopy architecture without substantially compromising the productive potential of wheat (Lemanowicz, 2023; Li et al., 2025b). Rather than functioning simply as growth-reducing chemicals, these compounds contribute to the development of a more mechanically stable crop canopy capable of supporting higher biomass under favourable growing conditions. Chlormequat chloride inhibits early GA-biosynthetic enzymes, such as copalyl diphosphate synthase and ent-kaurene synthase. Triazole compounds, like paclobutrazol and uniconazole, inhibit ent-kaurene oxidation in the microsomal fraction. Trinexapac-ethyl works later in the pathway by inhibiting GA-20-oxidase and GA-3-oxidase. This reduces the amount of active GA and preferentially shortens the upper internodes that contribute most to lodging (Rademacher, 2015; Zhang et al., 2021). The mode of action of different growth regulators is shown in **Figure 3**.

**Figure 3:**
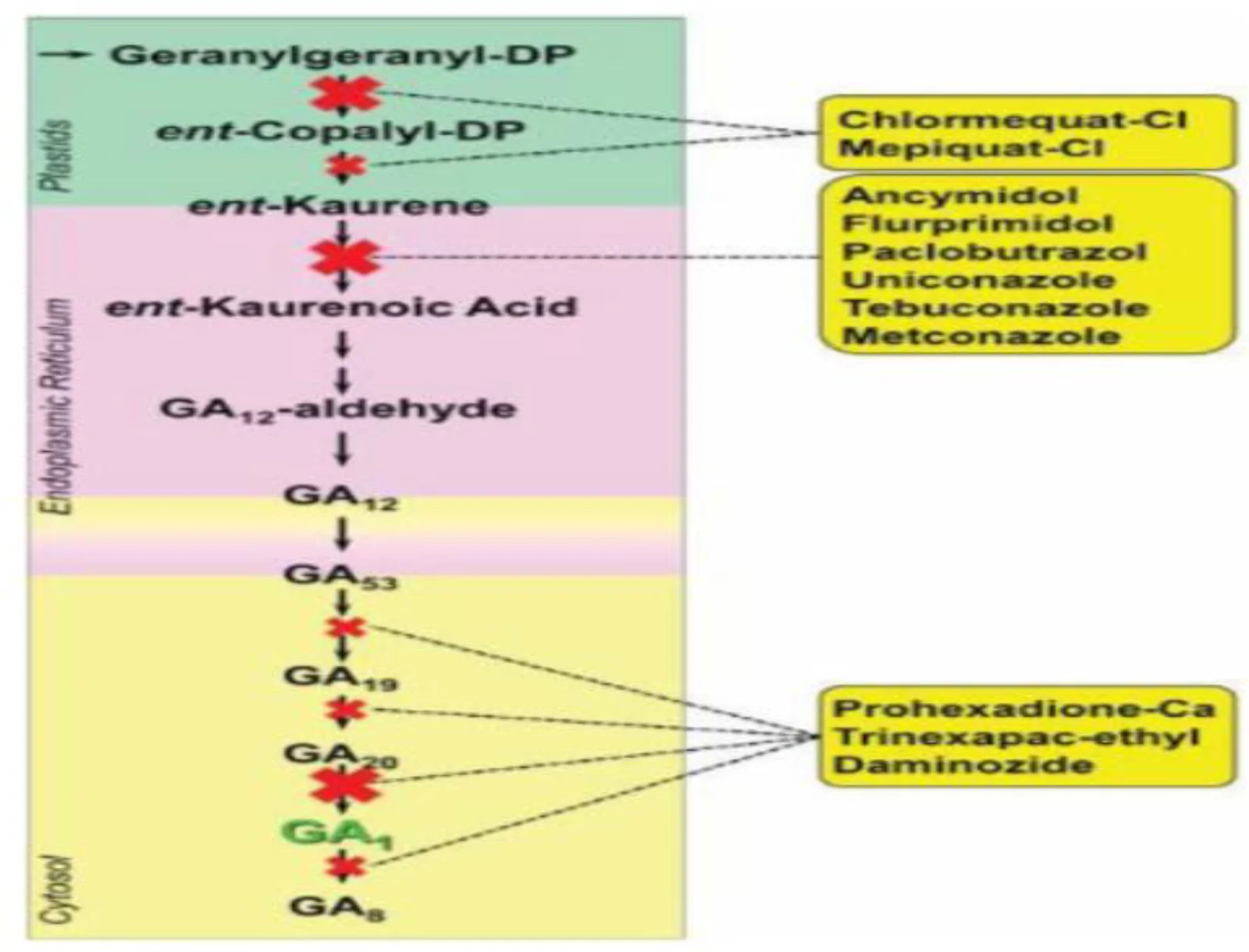
Sites of action of growth regulators in gibberellin biosynthesis pathway.

### Chlormequat chloride - early gibberellin inhibition

The use of chlormequat chloride (CCC) has become common in wheat production because it helps manage lodging by suppressing the early stages of gibberellin biosynthesis. When gibberellin activity is reduced, excessive internode elongation is limited (Wu et al., 2022). As a result, greater assimilate availability for stem development can contribute to thicker culms and reduce the likelihood of lodging in high-input production systems. CCC treatment often results in reduced plant height and increased stem diameter, which increases mechanical resistance against bending forces caused by wind or rain (Wu et al., 2022). In field experiments with soft white winter wheat, CCC applied at key developmental stages (e.g., stem elongation) consistently reduced stem height without negative effects on yield components, indicating its value in lodging management programs (Qin et al., 2020). Lodging percentage increased sharply with increasing nutrient level, rising from 2.9 per cent under control nutrition to 53.88 per cent at 200 per cent RDF with water spray. Application of growth regulators significantly reduced lodging at all nutrient levels, as shown in **Figure 4**. The lowest lodging was recorded under the combined application of CCC + tebuconazole, with lodging declining to 37.69 per cent at 200 per cent RDF and the overall mean decreasing to 23.34 per cent, compared with 32.35 per cent under water spray. This clearly indicates that CCC + tebuconazole effectively counteracted nutrient-induced lodging (Ghuman, 2021).

**Figure 4:**
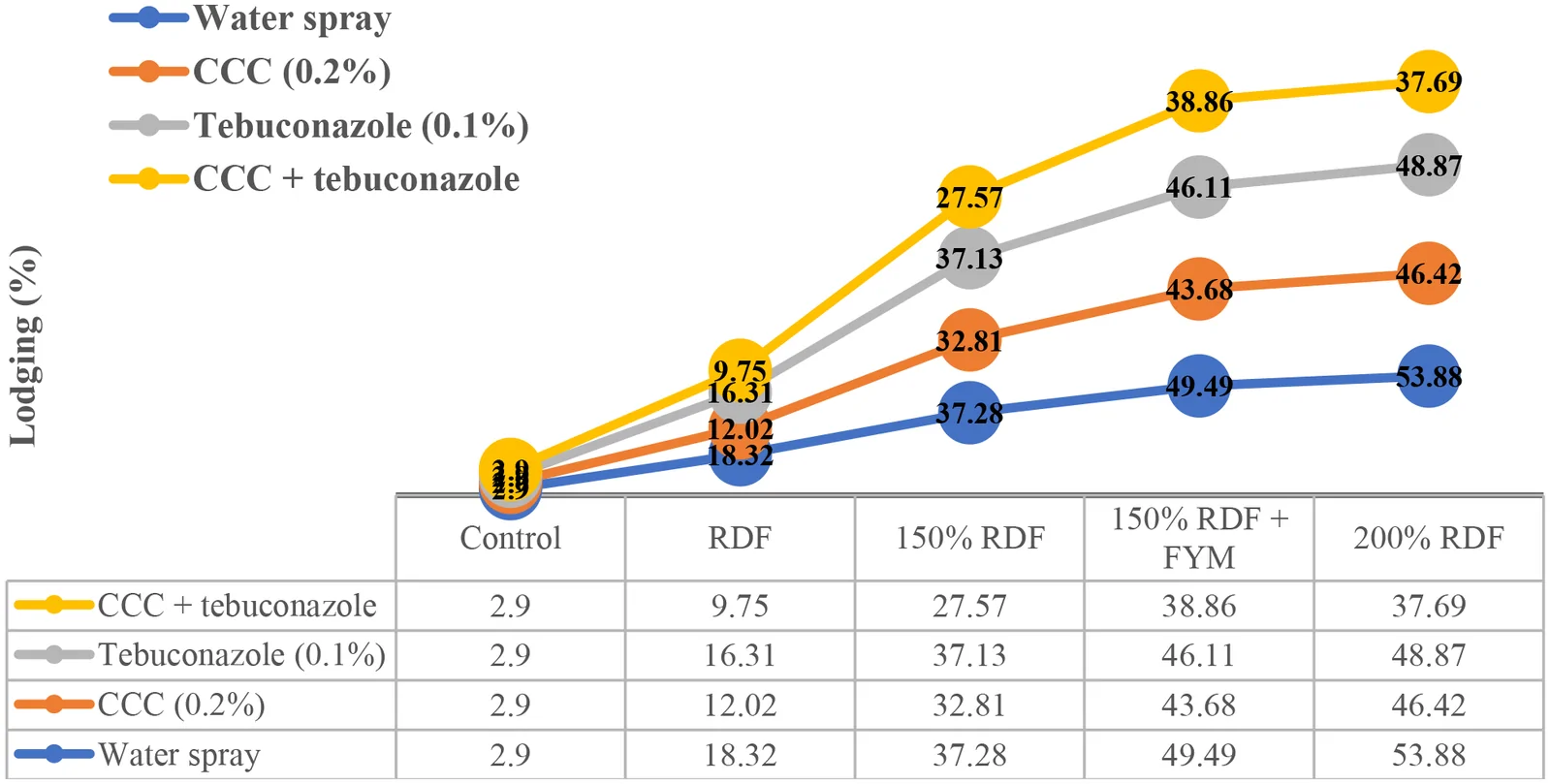
Lodging percent as influenced by different nutrient levels and plant growth regulators in wheat. Data taken from (Ghuman, 2021).

### Trinexapac-ethyl - targeted suppression of later GA biosynthesis

Trinexapac-ethyl (TE) is a cyclohexanedione-type PGR that acts by inhibiting late steps of gibberellin biosynthesis, resulting in significant reduction of internode elongation and enhanced stem sturdiness (Matysiak, 2006). Application of TE has been shown to decrease both total plant height and the length of upper internodes, which lowers the center of gravity and enhances lodging resistance in wheat (Matysiak, 2006). Studies conducted across multiple production environments have shown that trinexapac-ethyl (TE) can increase basal stem girth and contribute to greater plant stability during lodging-prone growth stages. Additionally, reductions in lodging following TE application have been associated with greater stability of wheat grain yield under intensive production conditions (Wiersma et al., 2011). For example, TE treatments produced shorter internodes and higher spike density without recorded lodging even under high nitrogen and dense stands. Trinexapac-ethyl inhibits the late steps of GA biosynthesis and preferentially shortens upper internodes without causing excessive growth suppression. TE generally increases stem strength, reduces lodging index and maintains grain yield under high nitrogen fertilization, making it suitable for intensive production systems (Kurepin et al., 2014; Wang et al., 2022). Trinexapac-ethyl application slightly reduced plant height from 91.4 cm in the control to a minimum of 85.4 cm at 100 g a.i. ha^-1^ applied at Feekes 7 (F7), and was accompanied by a small improvement in lodging rating from 1.2 (control) to 1.1 under higher-rate treatments. Grain yield increased modestly over the control (5.22 t ha^-1^) and reached the highest value of 5.41 t ha^−1^ at 100 g a.i. ha^-1^ at F7, indicating that a higher dose applied at the later stage slightly shortened plants, reduced lodging tendency, and produced the greatest yield advantage (Subedi et al., 2021).

### Uniconazole - dual effect on GA and structural reinforcement

Uniconazole is another GA biosynthesis inhibitor that, like TE, reduces plant height and internode elongation but can also influence cell wall composition and vascular development when applied at stem elongation stages (Li et al., 2025b). In comparison with CCC and some newer growth regulators, uniconazole was found to be more effective in strengthening basal stem tissues through enhanced lignin-related enzyme activity, which contributed to better lodging resistance rather than merely reducing plant height (Li et al., 2025b). While height suppression remains important, the additional biochemical reinforcement associated with uniconazole distinguishes its mode of action from CCC in agronomic practice. Uniconazole decreased internode length but increased internode diameter and plumpness, as shown in the **Figures 5**–**7**. These changes reflect thicker and stronger culms contributing to lodging tolerance. Uniconazole significantly increased grain yield, with the highest yield recorded under US2 (6.9 t ha^-1^) compared with the control (5.3 t ha^-1^). Yield further improved when combined with micronutrients, with UMS2 (7.7 t ha^-1^) being the highest (Ahmad et al., 2020).

**Figure 5:**
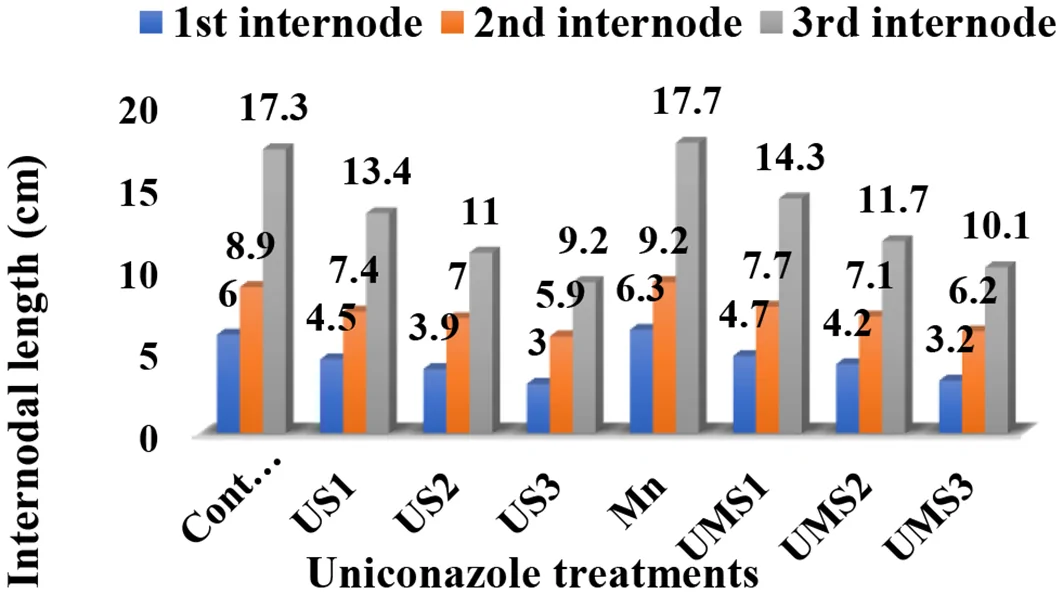
Effect of uniconazole (US) and uniconazole + micronutrient (UMS) treatments on the internode length (cm) of wheat. Data taken from (Ahmad et al., 2020).

**Figure 6:**
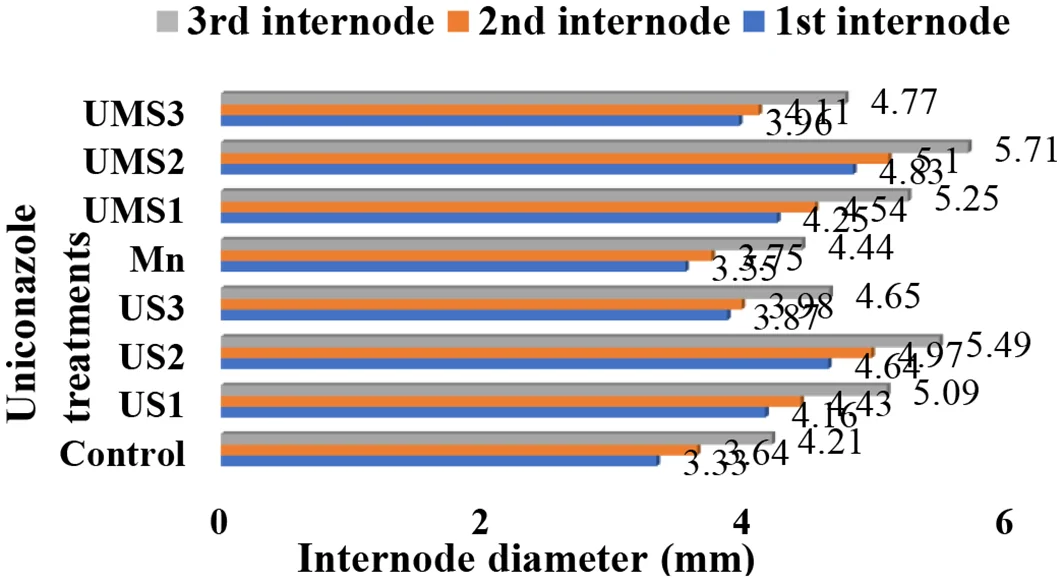
Effect of uniconazole (US) and uniconazole + micronutrient (UMS) treatments on the internode diameter (mm) of wheat. Data taken from (Ahmad et al., 2020).

**Figure 7:**
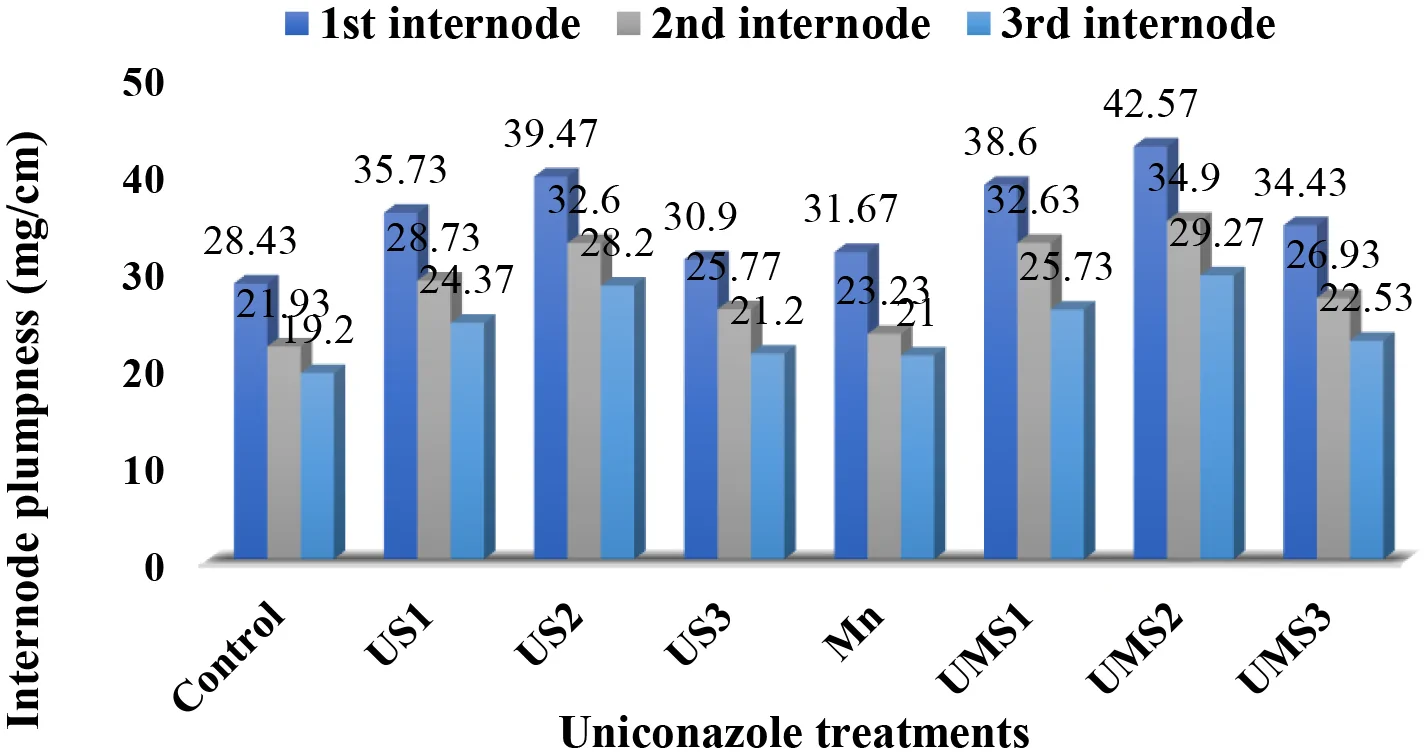
Effect of uniconazole (US) and uniconazole + micronutrient (UMS) treatments on internode plumpness (mg/cm) of wheat. Data taken from (Ahmad et al., 2020).

### Paclobutrazol - triazole regulator influencing internode strength

Paclobutrazol (PBZ) is a well-established triazole plant growth regulator that inhibits gibberellin biosynthesis and has been used to manage lodging by restricting stem elongation and increasing stem mechanical strength (Kamran et al., 2018). In wheat, paclobutrazol has been reported to increase basal culm diameter, culm filling and stem-wall development, thereby contributing to greater stem mechanical strength and lodging resistance. Evidence from rice has also shown similar effects of paclobutrazol on stem structure and lignification; however, these findings should be considered as comparative evidence rather than direct evidence for wheat (Kamran et al., 2018). Paclobutrazol shortened internodes and lowered the centre of gravity height, as shown in **Figure 8**, resulting in reduced plant height and better stability. At the same time, lignin content increased in culm tissues under PB treatments (**Figure 9**), further strengthening stems and contributing to lodging resistance. Yield increased up to PB 200 mg L^-1^ and then declined at higher concentrations, indicating that moderate paclobutrazol dose was optimal.

**Figure 8:**
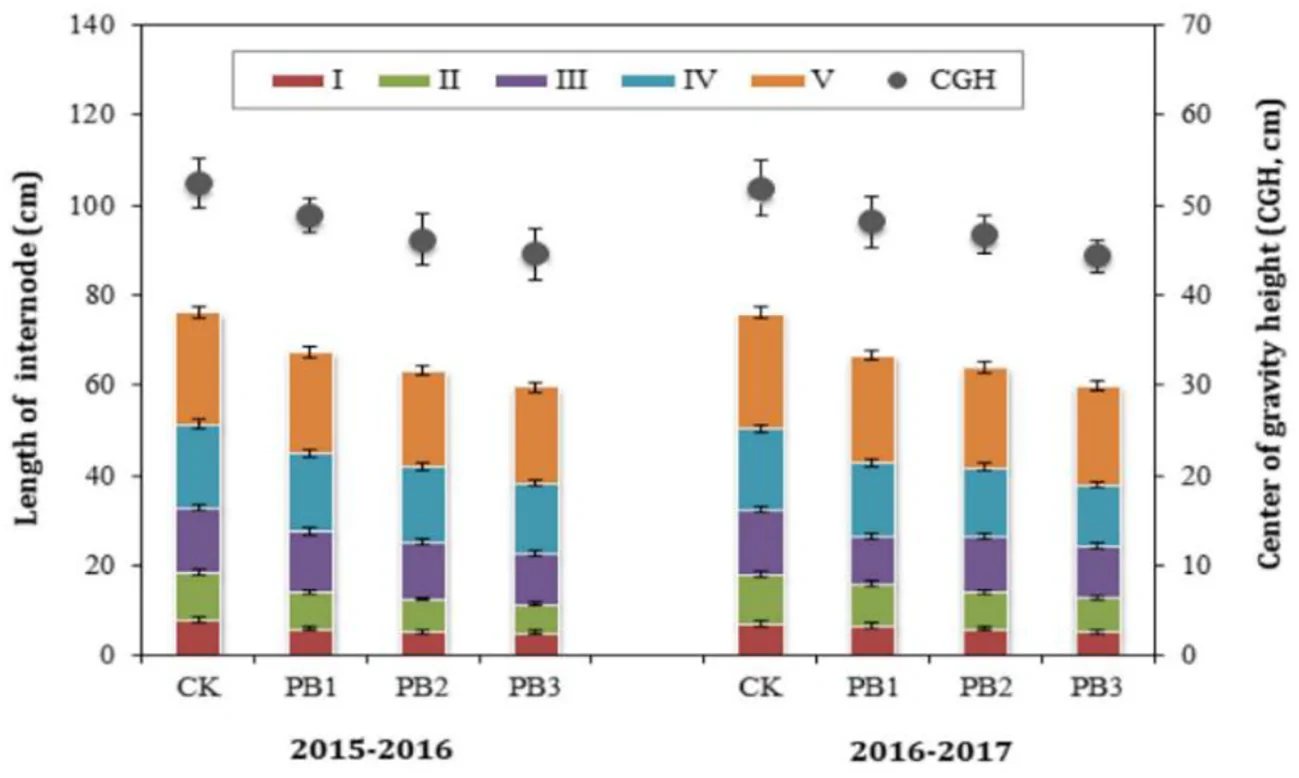
Effect of paclobutrazol on the length of individual internodes and center of gravity height of wheat. Adapted from (Kamran et al., 2018).

**Figure 9:**
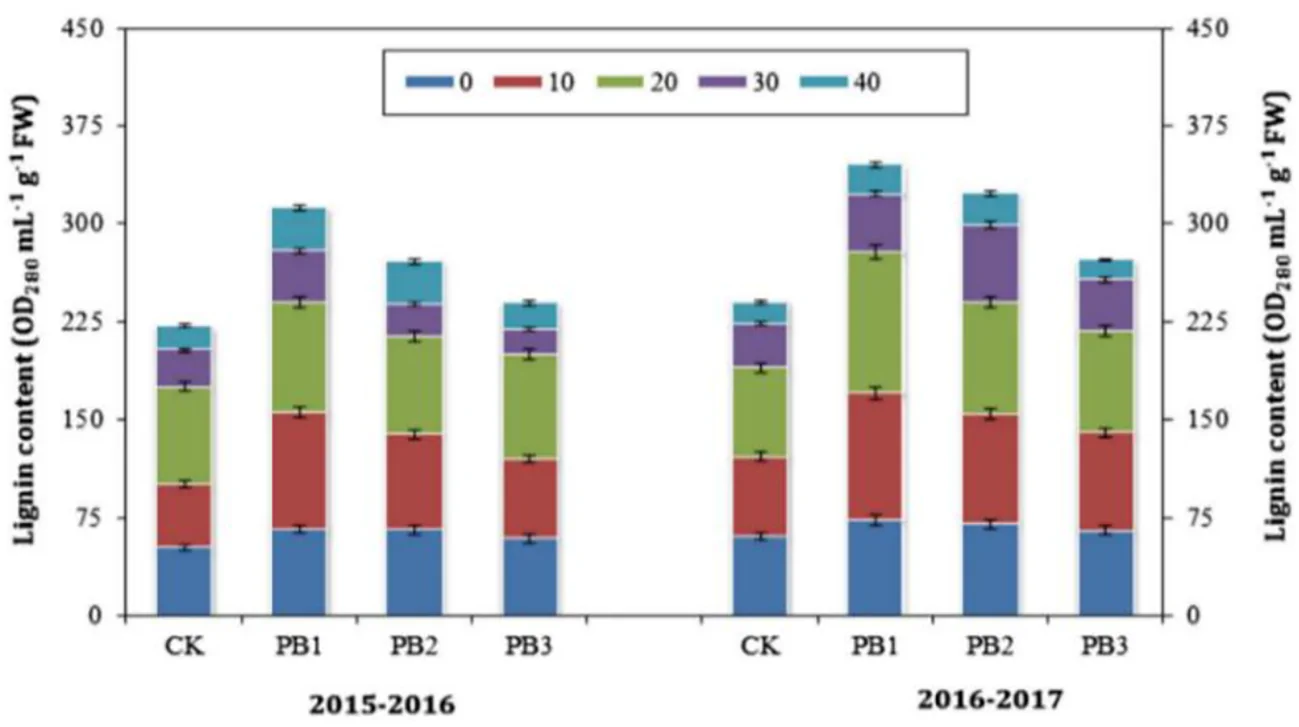
Effect of paclobutrazol on the lignin accumulation of wheat basal second internode. Adapted from (Kamran et al., 2018).

### Ethephon - ethylene release and stand structural effects

Ethephon is a compound that releases ethylene and modifies stem elongation and canopy structure to reduce lodging. Upon application, ethephon decomposes within the plant to release ethylene, which inhibits cell elongation in the upper internodes, increases culm wall thickness, and accelerates leaf senescence, thereby lowering the centre of gravity of the canopy. Application of ethephon to wheat typically produces shorter plants with shorter upper internodes, greater stem-breaking strength, and improved lodging resistance (Berry et al., 2004b; Rajala and Peltonen-Sainio, 2002). Ethephon can also alter assimilate distribution by enhancing remobilization of carbohydrates from stems to developing spikes, which may stabilize grain yield when lodging pressure is high. The adverse effects of inappropriate ethephon application, particularly at excessive rates or too early in crop development, have been documented and may include excessive plant shortening, spike sterility, and reduced grain yield (Zhu et al., 2016; Kang et al., 2014). As a result, optimizing timing and dosage of applications is necessary to achieve good results. When used under high nitrogen fertilizer applications and irrigation, ethephon has demonstrated an increased effectiveness for tall wheat varieties that are very susceptible to lodging. The interaction between ethephon dose and stage of application significantly influenced lodging index and grain yield, as presented in **Figures 10** and **11**. Higher ethephon rates applied at suitable stages markedly decreased lodging and were associated with improved yield performance.

**Figure 10:**
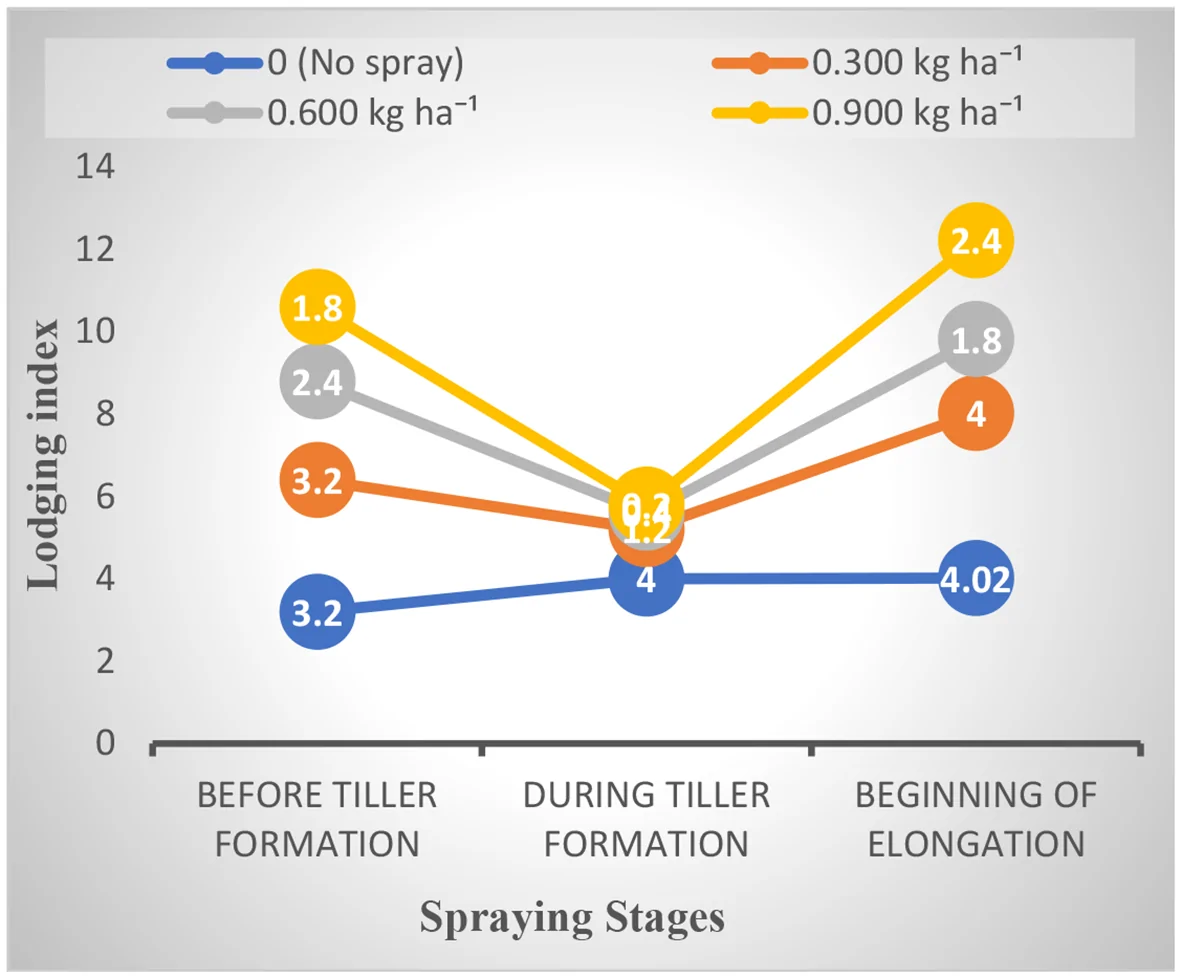
Interaction effect of ethephon levels and spraying stages on lodging index of wheat. Adapted from (Zaibel and Mohsen, 2022).

**Figure 11:**
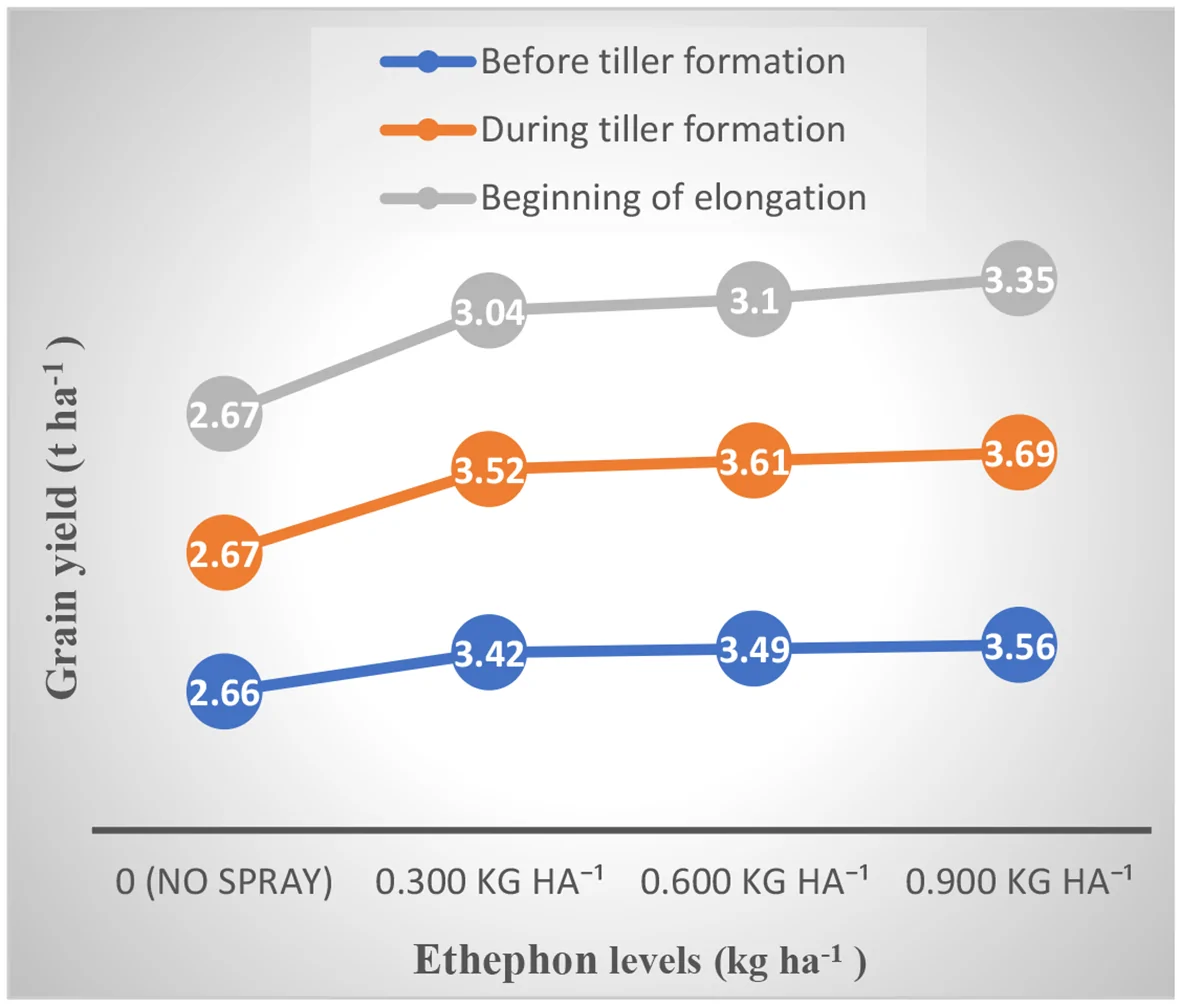
Interaction effect of ethephon levels and spraying stages on grain yield of wheat. Adapted from (Zaibel and Mohsen, 2022).

### Chlormequat chloride and uniconazole in lodging resistance

Chlormequat chloride (CCC) reduces plant height, shortens basal internodes and increases culm wall thickness by restricting gibberellin biosynthesis and modifying assimilate partitioning toward structural tissues. As a result, CCC application enhances stem breaking strength and reduces lodging susceptibility under high nitrogen input systems (Rajala, 2003; Zhang et al., 2020). CCC inhibits early steps in gibberellin biosynthesis and is widely used to reduce plant height and upper internode elongation, leading to a lower centre of gravity and therefore decreased lodging risk (Li et al., 2025b; Lemanowicz, 2023). Improvement in stem mechanical stability following uniconazole application has been linked not only to reduced stem elongation but also to greater lignification and thickening of culm tissues, which together enhance resistance against lodging (Li et al., 2025b; Ghuman, 2021). Compared with uniconazole, CCC produced greater suppression of plant height, whereas uniconazole contributed more strongly to the reinforcement of stem structure. The effectiveness of each regulator appeared to depend on cultivar characteristics and the type of lodging observed (Li et al., 2025b; Peng et al., 2025). The increases in culm breaking strength and decreases in lodging index following CCC and uniconazole (S3307) application were associated with alterations in endogenous hormone balance, including lower GA and IAA levels and higher ABA and cytokinin levels (**Figures 12**, **13**).

**Figure 12:**
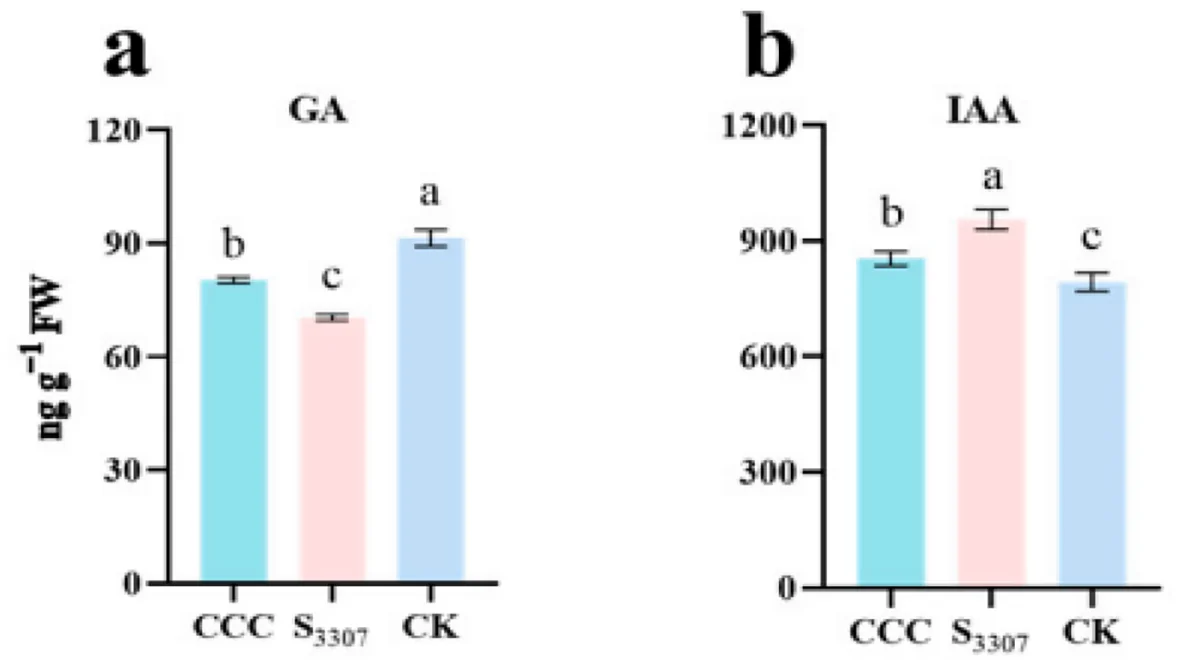
Effect of CCC and S3307 on plant hormones of the wheat at jointing stage. **(a)** GA; **(b)** IAA. Adapted from (Li et al., 2025b).

**Figure 13:**
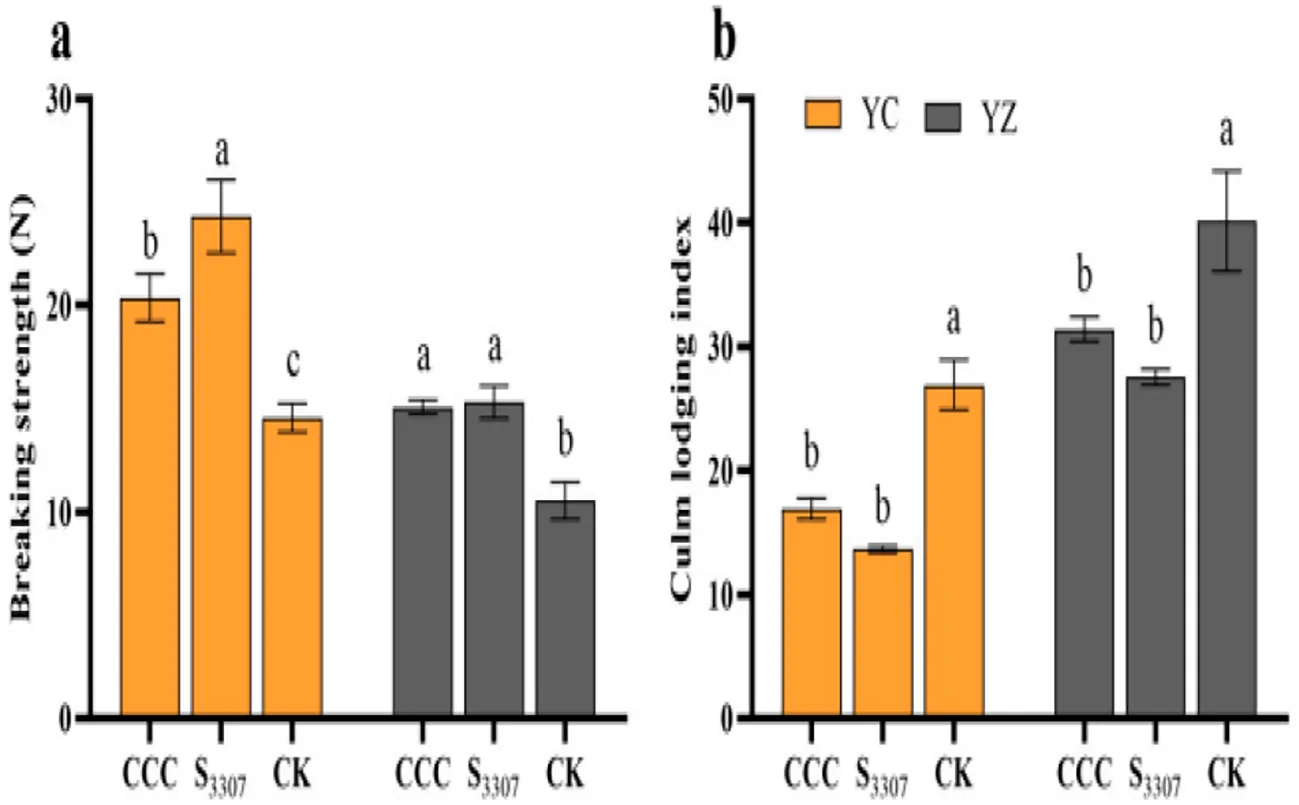
Effect of CCC and S3307 (uniconazole) on breaking strength and culm lodging index of wheat. **(a)** Breaking strength; **(b)** culm lodging index. Adapted from (Li et al., 2025b).

To operationalize these concepts under field conditions, a practical flow-based framework for selecting appropriate plant growth regulators and determining their optimum time of application is presented in **Figure 14**.

**Figure 14:**
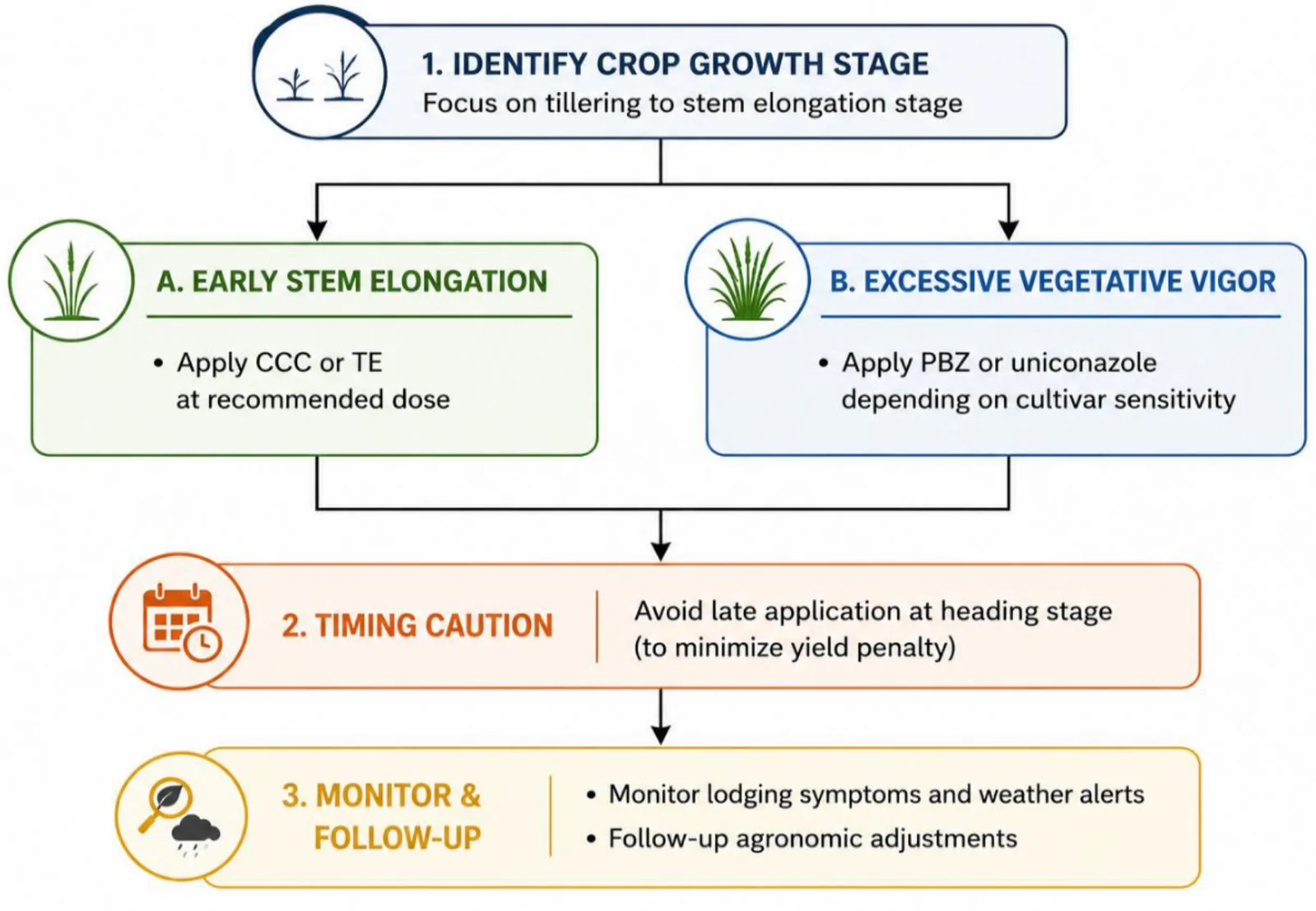
A practical flow-based framework for selecting the appropriate plant growth regulator and timing of application under field conditions.

The major plant growth regulators used in wheat lodging management act predominantly through modification of gibberellin or ethylene signalling pathways, resulting in altered internode elongation and culm biomechanics. Plant growth regulators used for lodging management in wheat differ considerably in their physiological action and field response. Compounds such as CCC, trinexapac-ethyl, paclobutrazol and uniconazole mainly act through inhibition of gibberellin biosynthesis, resulting in reduced stem elongation and improved culm strength, whereas ethephon influences plant growth through ethylene release and modifies elongation behaviour together with assimilate distribution. Although these regulators share the common objective of reducing lodging, they vary in biochemical mode of action, duration of activity, extent of height suppression, and interaction with nitrogen and moisture conditions, which ultimately lead to differences in lodging response among cultivars and production environments. A comparative overview of major plant growth regulators, including their recommended doses, application stages, physiological targets, modes of action, and lodging-related effects in wheat, is presented in **Tables 4** and **5**.

**Table 4:** Major plant growth regulators used for lodging control in wheat: modes of action, morphological effects and lodging outcomes.

Plant growth regulator	Primary hormonal/biochemical target	Main morphological effects	Effects on culm anatomy/strength	Overall effect on lodging
Chlormequat chloride (CCC)	Inhibits early steps of GA biosynthesis (Rademacher, 2015)	Reduces plant height and internode elongation (Rajala, 2003)	Increases stem wall thickness and culm diameter (Li et al., 2025b)	Decreases stem lodging (Zhang et al., 2014)
Trinexapac-ethyl (TE)	Inhibits GA 3β-hydroxylase (Zhang et al., 2014)	Shortens upper internodes and canopy height (Rajala, 2003)	Increases basal internode breaking strength (Zhang et al., 2014)	Strong reduction in stem lodging (Zhang et al., 2014)
Paclobutrazol (PBZ)	Triazole inhibitor of GA pathway (ent-kaurene oxidase) (Li et al., 2016)	Strong height reduction; compact plant type (Rademacher, 2015)	Increases lignin and cellulose deposition (Li et al., 2016)	Improves stem rigidity; reduces lodging (Li et al., 2016)
Uniconazole	Inhibits GA biosynthesis; affects ABA signaling (Rademacher, 2015)	Reduces elongation; darker, thicker leaves (Rajala, 2003)	Enhances culm wall thickness and vascular bundle area (Li et al., 2025b)	Improves mechanical strength and lodging resistance (Rademacher, 2015)
Ethephon	Releases ethylene in plant tissues (Koleva-Velcheva et al., 2012)	Reduces elongation; modifies tillering and spike traits (Koleva-Velcheva et al., 2012)	Promotes radial cell expansion in culm tissues (Koleva-Velcheva et al., 2012)	Variable effect; dose- and environment-dependent (Rajala, 2003)

**Table 5:** Recommended dose, timing and growth stage of application of plant growth regulators for lodging control in wheat.

Plant growth regulator	Typical dose range	Growth stage of application	Primary target effect	Lodging-related outcome
Chlormequat chloride (CCC)	750–1500 ppm or 1.0-2.0 kg a.i. ha^-1^ (Rajala, 2003; Rademacher, 2015)	Tillering to early stem elongation, Z30-Z32 (Berry et al., 2004b)	Suppresses GA-dependent internode elongation (Rademacher, 2015)	Reduced plant height and lodging index (Zhang et al., 2020)
Trinexapac-ethyl (TE)	0.2-0.4 kg a.i. ha^-1^ (Zhang et al., 2014)	Stem elongation to flag-leaf emergence, Z37-Z39 (Zhang et al., 2014)	Inhibits GA 3β-hydroxylase (Zhang et al., 2014)	Large reduction in stem lodging (Zhang et al., 2014)
Paclobutrazol (PBZ)	200–600 ppm or 50–200 g a.i. ha^-1^ (Li et al., 2016)	Early stem elongation Z30-Z32 (Li et al., 2016)	Inhibits ent-kaurene oxidase in GA pathway (Li et al., 2016)	Increased lignin/cellulose and culm strength (Li et al., 2016)
Uniconazole	50–150 g a.i. ha^-1^ (Rademacher, 2015)	Z31-Z37 early-mid stem elongation (Rademacher, 2015)	Inhibits GA; increases ABA signaling (Rademacher, 2015)	Improved culm wall thickness and lodging resistance (Rademacher, 2015)
Ethephon	150–300 ppm (Koleva-Velcheva et al., 2012)	Booting to heading Z45-Z55 (Koleva-Velcheva et al., 2012)	Releases ethylene; reduces elongation (Koleva-Velcheva et al., 2012)	Variable; depends on dose and environment (Rajala, 2003)

## Combined and optimized PGR strategies

Mixtures of different plant growth regulators may reduce lodging while requiring lower total application rates than when each product is used alone. Combining mepiquat chloride and PBC with an appropriate adjuvant has produced effective control of stem elongation and hormonal responses in winter wheat through the complementary effects of the two PGRs (Matysiak et al., 2025). Additional research has shown that combining MAP and PBC with appropriate adjuvants in winter wheat produced similar levels of growth regulation and lodging control while requiring a lower total PGR dose than either PGR alone (Matysiak et al., 2025). These approaches may have potential for sustainable wheat production systems where PGR type and application timing need to be adjusted according to cultivar, nitrogen management and environmental conditions.

## Seed priming versus foliar application of plant growth regulators

Plant growth regulators (PGRs) can be applied to wheat either through seed priming or foliar spraying. Seed priming with regulators such as paclobutrazol or uniconazole will be absorbed by the plant during germination and early seedling growth. Subsequently, the PGR will continue to provide hormonal regulation, inhibit gibberellin activity, and encourage a more compact plant structure. Typically, a plant grown from primed seed will produce shorter and stronger culms, create a thicker basal node, anchor better with its roots, and accumulate more lignin than an untreated plant (Zhang et al., 2019; Hussain et al., 2021). Biochemically, priming can lead to early increases in PAL, CAD and POD enzyme activity in the basal stem tissue, which will also contribute positively to strengthening the stem as the crop continues to develop (Ma et al., 2021). PGRs are commonly used as foliar applications at the tillering, stem elongation, or flag leaf stage of wheat development to effectively regulate and control canopy growth in the field under normal agronomic conditions. Foliar applications of CCC or trinexapac-ethyl are reported to reduce plant height, shorten the upper internodes, and increase stem wall thickness, thereby decreasing lodging index without significantly affecting yield when applied at optimal times (Wang et al., 2022; Niu et al., 2023). In contrast to seed priming, foliar application of PGRs primarily affects canopy structure and assimilate allocation during active vegetative growth rather than during the early stages of plant development. Therefore, seed priming with PGRs can enhance early stem strengthening and related biochemical processes, whereas foliar applications regulate plant height and internode elongation later in the crop growth cycle.

## Agronomic and integrated management strategies for lodging control

### Nitrogen management and lodging resistance

Plant height, basal internode length, and centre of gravity increase with excess nitrogen fertilization. These traits, which are predictive of a higher lodging index, are closely correlated with increasing amounts of nitrogen applied to the wheat crop (Li et al., 2022). While nitrogen improves the length of plant stems, it also decreases the density of basal internodes and the amount of cell wall components within each basal internode, thereby decreasing lodging resistance; conversely, moderate amounts of nitrogen combined with appropriate irrigation improve the thicknesses of the culm walls and their breaking strength (Li et al., 2022). Recent evaluations of wheat’s response to varying levels of nitrogen in the Huang-Huai-Hai Plain indicate that moderate nitrogen rates have improved lodging resistance for some wheat varieties, underscoring the need for accurate nitrogen management to enable grain production while minimizing lodging risk (Hu et al., 2025).

### Planting density and lodging susceptibility

Plant density influences the growth of wheat canopy cover and the strength of stems. Higher densities will increase the likelihood of competing against surrounding plants for light, often leading to taller, weaker stems with greater susceptibility to lodging (Chauhan et al., 2021). Maintaining an appropriate plant density can improve canopy uniformity and light distribution, contributing to stronger stem formation and more stable biomass partitioning and thereby reducing lodging risk in wheat (Zhang et al., 2023a).

### Irrigation and lodging dynamics

In addition, lodging behaviour of wheat is influenced by irrigation management since the water status impacts stem development and biomass in the canopy. High frequency and amount of irrigation can cause taller heights and higher biomass development that may increase bending force on the stem and lead to increased tendencies for lodging (Feng et al., 2023). On the other hand, proper irrigation management at certain growth stages like jointing and anthesis has been reported to enhance stem strength and breaking resistance (Feng et al., 2023).

### Root lodging and root anchorage

Root lodging occurs when the anchorage strength of the root-soil system is insufficient to withstand wind and canopy forces. Root plate spread, root biomass, and the strength and distribution of crown roots are important components of anchorage resistance in wheat (Berry et al., 2003; Piñera-Chavez et al., 2016). Wet or waterlogged soil can further weaken root-soil anchorage and increase the risk of root lodging, particularly under strong wind conditions (Berry et al., 2004b; Piñera-Chavez et al., 2016). Therefore, management practices that improve soil structure, rooting conditions, and drainage can contribute to lodging resistance. Conservation agriculture and reduced tillage can improve soil physical conditions and promote deeper and more extensive root growth in wheat, thereby potentially strengthening root anchorage (Mondal et al., 2019). Improving field drainage is also important where excess soil moisture weakens soil strength and root anchorage. In addition, plant growth-promoting rhizobacteria (PGPR) and biostimulants have potential to improve root architecture, root biomass, and root development, which may contribute to stronger plant anchorage under environmental stress (Dal Cortivo et al., 2017; Mohammed et al., 2022). Thus, improving root development and soil conditions should be considered alongside stem-strengthening approaches for comprehensive lodging management.

### Integrated agronomic-PGR strategies for lodging management in wheat

Lodging avoidance in wheat requires both improvement of plant structural properties and management of environmental conditions that favour lodging. Agricultural practices are used to manipulate canopy structure and plant nutrition and water supply, whereas plant growth regulators modify hormone balance and the growth properties of plant structures including stems and roots. Combining these two approaches is generally more effective for lodging management than using either approach independently, as agronomic practices can reduce canopy load while PGRs improve stem mechanical properties (Li et al., 2022a; Zhang et al., 2023a). Studies conducted recently have indicated that combining agronomic management and PGR treatment is more efficient compared to any individual method applied separately (Liu et al., 2023; Dai et al., 2023).

### Coordinated nitrogen management and growth regulation

Nitrogen significantly affects the tillering, canopy architecture, and elongation of internodes in wheat. High nitrogen levels usually lead to tall stature and weak stems which are prone to lodging, but low nitrogen levels hinder the development of yield components. This implies that nitrogen management and use of PGRs should be coordinated rather than done separately. Various studies have found that combining appropriate nitrogen levels with plant growth regulators such as CCC, trinexapac-ethyl, paclobutrazol, or uniconazole improves culm wall thickness, stem strength, lignin content, and lodging resistance compared with nitrogen management alone (Zhou et al., 2023; Zhang et al., 2024; Lemanowicz, 2023; Ghuman, 2021). Improved harvest index and bending resistance have also been observed in wheat under combined nitrogen and PGR management through several physiological mechanisms. Because high nitrogen stimulates gibberellin-mediated internode elongation, gibberellin-inhibiting PGRs are particularly effective in high-N systems (Makowski et al., 2020; Tripathi et al., 2022; Lollato et al., 2021). Split nitrogen applications synchronized with PGR timing reduce plant height and lodging index more effectively than either practice alone (Tripathi et al., 2022; Ma et al., 2023), whereas delayed or excessive topdressing may counteract regulator effects (Ma et al., 2023; Shah et al., 2019). Together, these findings demonstrate that nitrogen rate, timing and PGR scheduling must be coordinated with target canopy vigour and yield level (**Figures 15**, **16**).

**Figure 15:**
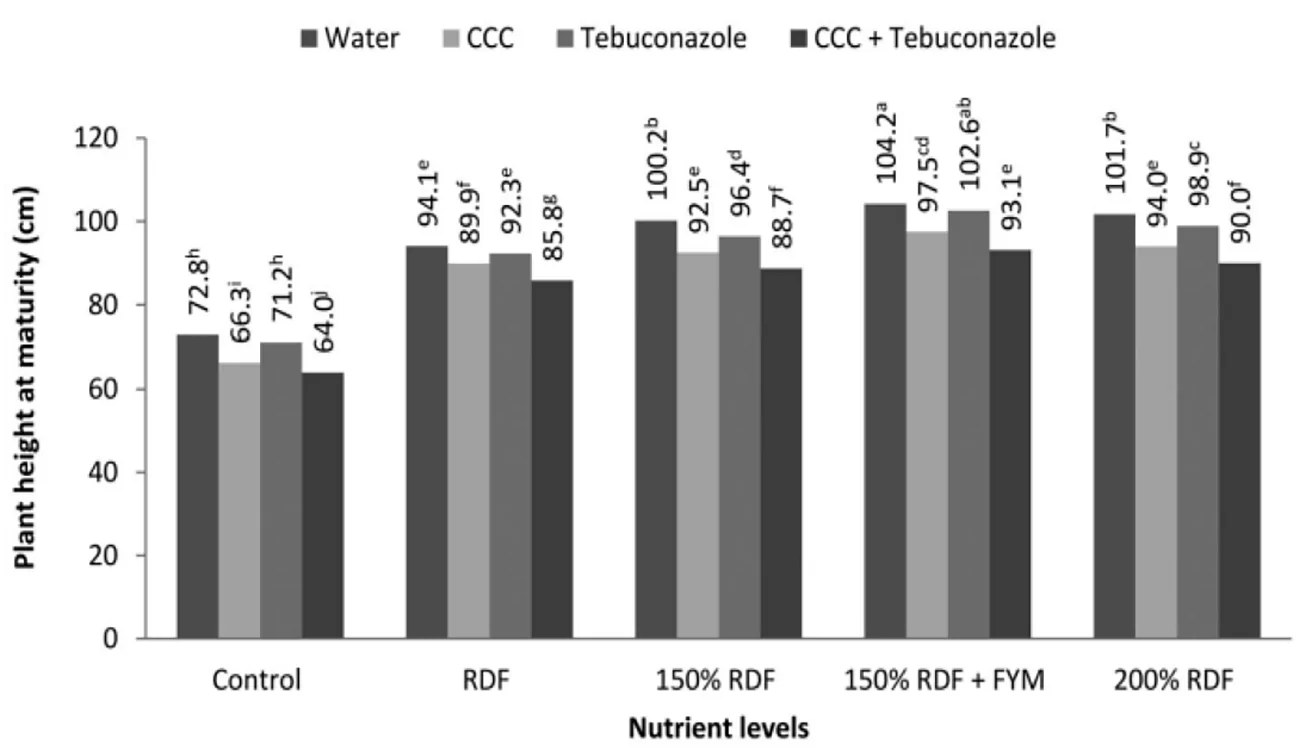
Interaction effect of nutrient levels and plant growth regulators on plant height at maturity of wheat. Adapted from (Ghuman, 2021).

**Figure 16:**
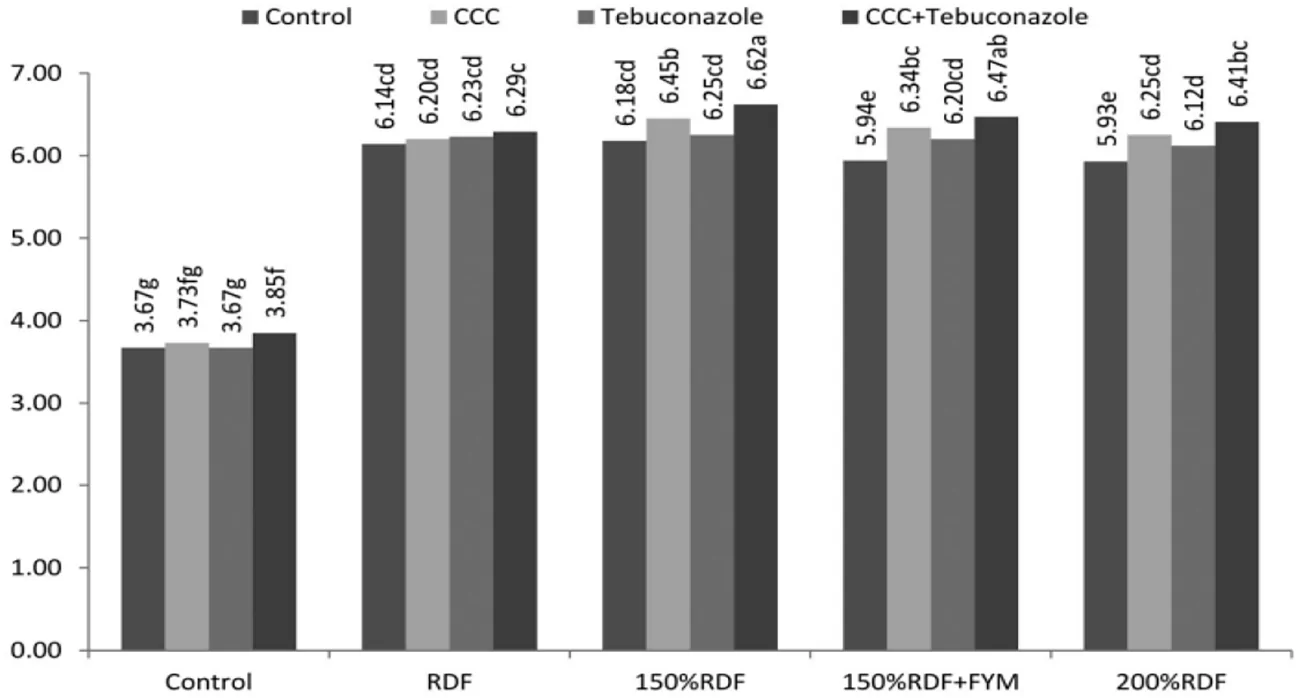
Interaction effect of nutrient levels and plant growth regulators on grain yield of wheat. Adapted from (Ghuman, 2021).

### Water availability, irrigation scheduling and PGR responsiveness

Water availability modifies biomass accumulation, stem biomechanics and root anchorage, thereby influencing lodging responses to PGRs. Under irrigated, high-biomass conditions, tall canopies and heavy ears increase stem and root lodging risks, and PGR use strengthens culm structure and reduces leverage forces (Nehe et al., 2020; Tripathi et al., 2022; Ma et al., 2023). Frequent irrigation during rapid stem elongation intensifies elongation pressure; synchronizing irrigation with PGR application reduces plant height, increases culm density and decreases lodging index while improving water productivity (Khan et al., 2023; Ahmad et al., 2023). Under water-limited conditions, PGRs often reduce vegetative demand, transpiring area and plant height, thereby enhancing drought tolerance and lowering lodging risk (Ma et al., 2023; Kendal et al., 2021; Lollato et al., 2021). However, excessive suppression during severe drought may restrict leaf expansion and grain set, indicating the need to adjust regulator dose to moisture regime (Kendal et al., 2021; Lollato et al., 2021). Overall, these results support environment-specific PGR scheduling embedded within irrigation planning (Nehe et al., 2020; Ma et al., 2023).

### Sowing time, plant density and establishment strategy in relation to PGRs

Wheat responses to PGRs are significantly influenced by sowing date and crop establishment because canopy architecture is largely established before stem elongation. Early sowing may extend the vegetative growth period, resulting in taller plants and greater lodging risk, whereas moderately delayed sowing can produce shorter and more structurally stable plants (Mondal, 2020). Early-sown and high-density wheat crops treated with CCC and trinexapac-ethyl have been shown to reduce excessive stem elongation while increasing culm wall thickness and stem strength, which improves lodging resistance without significant yield loss (Chen et al., 2023; Liu et al., 2023; Peng et al., 2025). Yield benefits from PGR use are therefore more evident in high-vigor early-sown wheat crops, whereas responses tend to be limited in late-sown shorter crops (Rahman et al., 2022). Since wheat phenology and node initiation are influenced strongly by temperature, the same application date may correspond to different developmental stages under different sowing windows. Consequently, PGR application in wheat should be based on crop phenological stage rather than fixed calendar timing (Wang et al., 2023; Zhang et al., 2023a; Shah et al., 2019; Lollato et al., 2021). These observations emphasize the importance of coordinating sowing date, plant population and PGR timing for effective lodging management.

### Cultivar architecture and genotype-specific integration of agronomy and PGRs

The effectiveness of growth regulation in wheat depends greatly on cultivar architecture and genotype-specific biochemical responses. Tall wheat cultivars that are highly susceptible to lodging generally respond more favourably to PGR programs integrated with suitable nitrogen management, irrigation scheduling and crop establishment practices, whereas semi-dwarf cultivars often require comparatively milder interventions (Wang et al., 2024). Recent studies further indicate that wheat cultivars differ in their physiological and biochemical response to PGRs, suggesting that cultivar-specific recommendations are more appropriate than generalized management strategies (Jat et al., 2025; Peng et al., 2025).

## Quantitative synthesis of lodging-related responses in wheat

Scientific literature has demonstrated that the efficacy of plant growth regulators on reducing lodging in wheat is affected by cultivar, agronomic practices and seasonal conditions. However, several consistent trends have been observed. For example, chlormequat chloride (CCC) typically reduces plant height by 8–18 per cent, with the largest reductions occurring when CCC is applied at the beginning of the stem elongation period. The degree of height reduction is also affected by nitrogen supply and cultivar sensitivity (Niu et al., 2023). Trinexapac-ethyl (TE) differs from CCC in its response because it inhibits gibberellin biosynthesis at a later stage, thereby reducing the development of upper internodes that contribute strongly to lodging susceptibility. As a result, TE usually increases lodging resistance more than one would expect based on height alone (Wang et al., 2022). Paclobutrazol often produces the greatest reduction in plant height compared with CCC and TE, with reductions commonly ranging from 20 to 35 percent. Reports indicate that inappropriate application timing or rates may cause excessive inhibition of vegetative growth; therefore, careful management of paclobutrazol is critical (Kamran et al., 2018). Research on lodging has also suggested that improving stem strength plays a greater role in lodging resistance than lowering height alone. TE applications have been associated with an approximately 30–55 per cent reduction in lodging index, compared with a 25–50 per cent reduction under CCC application These responses have been associated with thicker basal internodes and greater culm wall density, both of which enhance stem rigidity (Wang et al., 2022). Likewise, paclobutrazol has been reported to increase culm breaking strength by approximately 20–40 per cent, mainly because lignin and cellulose accumulate to a greater extent in the basal stem tissues, resulting in stronger structural support (Kamran et al., 2018).

The effects of plant growth regulators on wheat yield vary considerably across growing environments. In many experiments conducted under high nitrogen fertilization and irrigated or high-rainfall conditions, PGR application either maintained or slightly improved grain yield when lodging reduction occurred. Nearly 60–70 per cent of reported studies indicate neutral or positive yield responses under such situations (Lodhi et al., 2024). However, negative effects on wheat yield following plant growth regulator application have also been reported under certain conditions, particularly when excessive growth suppression occurred, drought stress followed application or naturally short-statured cultivars were treated (Berry et al., 2004b; Ahmad et al., 2023). These findings indicate that regulator type, application timing and dose must be adjusted carefully according to cultivar architecture and expected seasonal conditions. Comparisons among wheat cultivars further demonstrate that modern semi-dwarf genotypes generally possess lower lodging incidence and greater stem strength than older tall cultivars under comparable management conditions. Studies have reported nearly 15–35 per cent lower lodging occurrence together with 10–25 per cent higher stem breaking strength in modern cultivars because of long-term selection for standability traits (Huang et al., 2023). Nevertheless, some high-yielding wheat cultivars with larger spikes or excessive biomass may still remain susceptible to lodging under high nitrogen input or late-season wind and rainfall conditions, indicating that reduced plant height alone is insufficient for complete lodging protection. Overall, quantitative evidence suggests that effective lodging reduction in wheat depends on improving culm mechanical strength while moderately controlling plant height rather than excessively suppressing stem elongation. Meta-summary patterns further indicate that combining lodging-tolerant wheat cultivars with balanced nitrogen management and carefully selected PGR programs can reduce lodging incidence by nearly 40–70 per cent under high-risk production environments while also stabilizing grain yield performance (Lodhi et al., 2024).

## Case studies of wheat lodging: India, China and Europe

Lodging behaviour in wheat varies considerably across geographical regions because weather patterns, crop management practices, and cultivar use differ among production systems. In India, lodging frequently occurs in the Indo-Gangetic Plains when heavy seasonal rainfall coincides with high nitrogen fertilization and irrigated wheat production, particularly during critical reproductive stages. Substantial yield losses have been reported in these high-yielding environments when these conditions occur simultaneously. Field observations from Punjab and Haryana have reported lodging incidence ranging from approximately 15–40 per cent in late-sown wheat exposed to excessive nitrogen fertilization and humid conditions during later growth stages. This high incidence highlights the importance of integrating agronomic and cultivar-based approaches to lodging management in Indian wheat systems.

The occurrence of wheat lodging differs considerably among production regions because crop management practices, climatic conditions and cultivar characteristics vary widely. In the North China Plain (NCP), where intensive wheat-maize double-cropping systems are supported by high nitrogen inputs and dense plant populations, lodging remains a recurring production constraint. These management practices encourage vigorous vegetative growth but often reduce stem stability, increasing the likelihood of lodging, particularly under adverse weather conditions. Recent UAV-based surveys conducted in commercial wheat fields of Hubei Province successfully mapped lodging over areas larger than 2 km² and showed that lodging was unevenly distributed across fields, with severe damage concentrated in patches following rainfall events (Zhang et al., 2023b). European investigations have reached similar conclusions, although the factors driving lodging differ among environments. Comparative cereal studies indicate that lodging tolerance can be strongly influenced by interactions between genotype and local environmental conditions rather than by cultivar characteristics alone. In wheat, lodging risk varies among production environments according to differences in rainfall, crop management and cultivar characteristics, while studies in other cereals provide complementary evidence that genotype × environment interactions can substantially affect lodging susceptibility (Tumino et al., 2017).

## Remote sensing and UAV methods for wheat lodging detection

Recent advances in remote sensing technologies provide powerful tools for detecting, mapping and quantifying wheat lodging across field and regional scales. Unmanned aerial vehicles (UAVs) equipped with high-resolution RGB and multispectral sensors enable rapid, objective assessment of lodging incidence that overcomes many limitations of manual scoring in the field (Zhang et al., 2023b). UAV-based deep learning models, including convolutional neural networks, have achieved F1 scores above 90 per cent in classifying lodged vs non-lodged areas across heterogeneous fields of several square kilometres, demonstrating feasibility for large-scale monitoring and post-disaster damage assessment (Zhang et al., 2023b). Machine learning approaches are now being increasingly used for wheat lodging detection from UAV imagery because they can identify canopy distortion more accurately than conventional image analysis methods. Deep learning architectures such as EfficientNet, ResNet and semantic segmentation models including Deeplabv3+ have shown good performance in distinguishing lodging severity levels using canopy inclination, orientation and lodging ratio as key image features (Azizi et al., 2024). In wheat, these systems can estimate lodged area percentage as well as average lodging angle, and repeated UAV flights further allow tracking of lodging progression over time rather than providing only single-time observations.

Satellite-based remote sensing has likewise been explored for assessing wheat lodging over much larger areas, especially for crop insurance and regional loss estimation programs. Detailed satellite imagery from platforms such as GaoFen-2, combined with deep learning-based semantic segmentation, has achieved F1 scores above 85 per cent for wheat lodging extraction (Tang et al., 2022). These approaches offer potential for regional-scale lodging assessment where UAV-based monitoring is impractical. Although satellite-based wheat lodging extraction has been successful, several challenges remain. These challenges arise from variation in plant height and changing illumination conditions between image acquisition dates. Lodging detection may also be constrained by the spatial resolution of satellite sensors. Continued improvement in algorithms, sensor fusion (RGB + multispectral + LiDAR) and integration with ground truthing will enhance the operational utility of remote sensing for lodging surveillance and decision-support systems in precision agriculture.

## Climate change, extreme weather and increasing lodging risk in wheat

Climate change is expected to increase the frequency of extreme weather events such as heavy rainfall, strong winds and storms, all of which intensify lodging incidence in wheat. Rising CO_2_ concentrations and warmer temperatures promote greater biomass accumulation and canopy height, which increases the center of gravity and mechanical leverage on basal internodes, enhancing susceptibility to stem and root lodging (Foulkes et al., 2011). Extreme rainfall during grain filling increases ear weight while simultaneously softening weakened stems, greatly amplifying lodging probability in high-input systems (Berry and Spink, 2012). Climate-driven increases in storm frequency and peak wind speeds during reproductive stages have already been associated with widespread lodging events in major wheat-growing regions (Rajkumara, 2008). Waterlogging caused by heavy rainfall can weaken root anchorage in wheat and increase the occurrence of root lodging, which differs from stem lodging in both mechanism and field expression (Setter and Waters, 2003). Such conditions are expected to become more frequent under changing rainfall patterns associated with climate variability. Consequently, even with improvements in wheat genetics and agronomic management, future climatic instability is likely to maintain or even intensify lodging risk in modern production systems.

## Limitations, risks and environmental issues

### Agronomic limitations and yield trade-offs

Although plant growth regulators are widely used for lodging control in wheat, their effects are not always beneficial under every production situation. Research on trinexapac-ethyl and nitrogen management in southern Brazil demonstrated that although higher rates of trinexapac-ethyl reduced lodging and maintained grain yield, seed quality declined under certain nitrogen levels (de Faria et al., 2022). This potential trade-off between lodging resistance and seed vigour may be associated with reduced seed quality under certain treatment conditions. Comparable results have been reported when PGRs were applied at inappropriate growth stages or at excessive rates (Singh et al., 2019). Such applications may cause excessive plant height reduction, smaller spikes and fewer grains, particularly under stressful growing conditions (Singh et al., 2019). Scientific evidence suggests that PGR applications in wheat should be adjusted according to cultivar characteristics, nitrogen management, and environmental stress conditions because uniform application strategies are not always beneficial.

### Uncertain responses under variable field conditions

Plant growth regulators (PGRs) do not produce consistent responses across wheat-growing regions because their effectiveness depends on interactions among crop genotype, site-specific environmental conditions, and agronomic management practices. Field trials conducted across different regions have demonstrated that the same PGR treatment can reduce lodging and improve grain yield in some environments, whereas in others it may have little positive effect or may even reduce yield (Espindula et al., 2009; Ghuman, 2021). These variable results illustrate that the performance of a regulator cannot be evaluated independently of the production environment. Multiple interacting factors during crop development contribute to variability in PGR performance. During stem elongation, soil nutrient availability, temperature and moisture regimes and cultivar-specific hormonal sensitivity influence the physiological response to PGRs and their effects on lodging resistance and yield (Espindula et al., 2009; de Faria et al., 2022). Because these factors can vary substantially among locations and seasons, a single PGR recommendation is unlikely to perform equally well across all production environments. Therefore, greater emphasis is being placed on developing PGR programs tailored to specific cultivars and agroecological environments, enabling better management decisions than generalized recommendations intended for all production conditions.

### Residues in grain and regulatory limits

Regulatory evaluation of plant growth regulators considers not only their agronomic efficacy but also their persistence in harvested grain and potential food-safety implications. The international regulatory authorities have evaluated chlormequat chloride extensively and have used that information on its metabolism, residue behaviour and toxicological profile to make recommendations on safe use and maximum residue limits (EFSA, 2009; FAO/WHO Joint Meeting on Pesticide Residues (JMPR), 2017). Although residue levels generally decline between application and harvest, detectable residues may remain depending on application timing and rate, crop management practices, and the pre-harvest interval; therefore, compliance with recommended pre-harvest intervals and maximum residue limits is an important component of responsible PGR use (FAO/WHO Joint Meeting on Pesticide Residues (JMPR), 2017; EFSA, 2025). Based upon currently available assessments, application of chlormequat chloride according to Good Agricultural Practice is not anticipated to result in unacceptable dietary exposure to humans (EFSA, 2025). However, changes in crop production practices over time may influence residue behaviour. Therefore, continued ongoing monitoring of grain residue is needed to confirm that current regulatory assessments will continue to be relevant as commercial production continues to evolve.

### Environmental fate and non-target impacts

The physicochemical properties of chlormequat chloride largely determine its environmental behaviour following field application. Chlormequat chloride has a high solubility in water and moderate mobility throughout the soil, which provides the potential for chlormequat chloride to move from the site of application into intermediate soil layers or adjacent surface waters, particularly with repeated applications (Guo et al., 2010; USEPA, 2021). Though chlormequat chloride is not a persistent contaminant based on its degradation characteristics, its potential for transport through the environment has prompted the development of environmentally sensitive analytical methods to monitor residues in surface and drinking water (USEPA, 2021). Chlormequat chloride has also been identified in ecological risk assessments as requiring careful management because of concerns regarding potential reproductive and developmental toxicity under certain exposure scenarios (PPDB, 2025). Similar issues have been raised regarding several of the triazole growth regulators; thus, greater attention has been placed on reducing application rates, enhancing active ingredient formulations, and integrating non-chemical methods into lodging management programs (Carvalho, 2017). The above-mentioned actions are consistent with an increasing preference for reducing environmental exposures while maintaining agricultural effectiveness.

### Sustainability and future regulatory pressure

Future use of plant growth regulators in wheat production will likely be influenced by changing regulatory policies and increasing emphasis on environmentally sustainable crop management. Numerous agricultural markets are now placing stronger emphasis on reducing synthetic chemical inputs and the implementation of more stringent residue standards, specifically for export-orientated production systems (Carvalho, 2017/EU EFSA, 2025). Increasing regulatory requirements may reduce reliance on routine PGR applications as producers shift towards more integrated approaches to lodging management. Lodging management is increasingly moving towards integrated production systems that combine resistant cultivars, balanced nitrogen nutrition, appropriate irrigation and optimized sowing time, with chemical regulators used when these measures alone are insufficient (Hu et al., 2025; Mondal, 2020). Advances in wheat breeding and future advancements in managing wheat crops are therefore anticipated to be more significant in reducing lodging risk than will the continued use of PGRs for the purpose of controlling lodging (Mondal, 2020; Shah et al., 2025). In this overall context, PGRs should be considered to be supporting management tools that are used to improve the performance of well-planned agronomic systems and not the primary mechanism for controlling lodging in wheat.

## Economic consequences of lodging in wheat

Economic losses due to wheat lodging arise from reduced grain yield and quality, as well as greater harvesting difficulty and higher production costs. It also leads to greater harvesting challenges and higher production costs. Reports from various wheat-growing areas show that lodging can cut grain yield by almost 10 to 57 per cent. The extent of this loss differs based on the type of cultivar, weather conditions, and management practices (Feng et al., 2023). Studies using proximal sensing and remote sensing technologies have found that serious lodging events in commercial wheat fields often lead to yield drops of about 25 to 30 per cent. In some major lodging events reported in China and other leading wheat-producing areas, yield losses of around 27 per cent or more occurred when large crop areas were impacted (Jiang et al., 2022). Research using image analysis shows that yield damage in wheat can be extremely severe if lodging happens before or during the early grain-filling period. Under these circumstances, losses of 40 to 60 per cent have been reported. This severe reduction occurs because stem collapse disrupts the canopy structure, decreases light capture, and limits the movement of nutrients to the developing grains (Yu et al., 2022; Wang et al., 2023). At the regional scale, analyses of yield constraints in farmers’ fields have shown that lodging during reproductive stages can account for around one-third of total yield loss, with one survey attributing approximately 38 per cent of yield loss to reproductive-stage lodging relative to other stress factors such as pests, weeds and poor stand establishment (Chauhan et al., 2018). These findings demonstrate that lodging is a major yield-limiting factor in modern high-input wheat systems. Economic consequences are not confined to yield quantity; quality penalties are also substantial. Lodged crops experience increased contact between spikes and moist soil, higher canopy humidity, slower drying and greater exposure to rainfall events, all of which predispose grain to shrivelling, pre-harvest sprouting and fungal infection. Recent spectral and imaging studies in Indian wheat fields have reported deterioration in key quality attributes, including grain brightness and protein-related indices, with increasing lodging severity, indicating that quality losses accompany yield losses in lodged stands (Sharda et al., 2025). Nitrogen lodging interaction studies in different gluten types show that too much nitrogen raises the risk of lodging. It can also lower baking and end-use quality. On the other hand, nitrogen treatments that improve lodging resistance typically support more stable grain quality profiles (Hu et al., 2025). Thus, lodging can result in market price discounts through reduced grain quality in addition to direct yield losses.

Harvest operations represent another major source of economic impact. Lodged wheat is more difficult to cut and feed into combines, requiring slower travel speeds, lower cutter-bar height and more passes, which together elevate fuel use, machine wear and labour costs. Recent reviews of cereal lodging emphasise that lodged fields are routinely harvested less efficiently, with harvest losses and spillage substantially higher than in standing crops, and that the extra time required increases the risk of further weather damage before harvest can be completed (Niu et al., 2022b; Shah et al., 2022). Remote-sensing based assessments also highlight the importance of rapid lodging mapping for insurance claims and disaster response, underscoring that lodging has recognized financial implications at the farm and policy levels beyond the agronomic domain (Jiang et al., 2022).

When yield losses, quality downgrades and higher harvesting costs are considered together, the net income impact can be severe. Economic and agronomic evaluations of integrated nitrogen-PGR management indicate that, where lodging is common, treatments that reduce lodging frequency and severity can provide higher gross returns and benefit-cost ratios than unmanaged high-nitrogen controls, even after accounting for PGR costs (Malkarnekar and Singh, 2023). Systems-level analyses of resilient wheat production in subtropical agro-ecosystems similarly indicate that agronomic packages which lower lodging risk-through improved establishment, residue management and canopy control-tend to deliver 5–20 per cent higher yields and 10–30 per cent higher profitability, especially in seasons with frequent storms or heavy rainfall (Jat et al., 2025; Khan et al., 2023). In this context, preventing lodging is best viewed as a risk-management investment: it protects yield potential, maintains grain quality, preserves harvesting efficiency and stabilizes farm income under increasingly variable climatic conditions.

## Critical discussion and research controversies

Although there has been major progress in understanding lodging resistance in wheat, several issues continue to be debated because results are not always consistent between different environments and management systems. For example, while PGRs consistently induce reduced wheat plant height, the relationship between plant height and lodging resistance is more complex than previously considered. Increasingly, lodging resistance in wheat is considered to be more closely related to stem strength than to plant height alone. The last few years of research indicate that similar height plants may differ considerably in their ability to remain erect due to differences in the mechanical properties of the culm. Increased basal internode thickness, stronger culm tissues and greater stem rigidity have all been consistently associated with greater lodging tolerance. Supporting this view, Peng et al. (2025) demonstrated that the structural characteristics of the basal internodes are fundamental to the ability of wheat plants to withstand external mechanical forces. Their research suggests that plant stature and stem morphology should be evaluated together when evaluating lodging resistance. This same principle is demonstrated in the performance of PGRs; although different PGR compounds may produce similar reductions in plant height, their impact on stem anatomy and the mechanical reinforcement of the stem may differ greatly. This indicates that similar reductions in plant height do not necessarily result in equivalent lodging control among different PGRs. Hence, PGR effectiveness depends on their effects on stem characteristics rather than on height reduction alone. Another source of variability is nitrogen management as well. Increasing nitrogen availability will usually stimulate growth but optimal rates of fertiliser for both high crop yield and lodging resistance vary by cultivar and production conditions. For example, Hu et al. (2025) reported that precise nitrogen management improved lodging resistance at the Huang-Huai-Hai Plain without negatively affecting grain yield. However, consistent benefits for lodging resistance have not been demonstrated across contrasting soil types and climates, indicating that nitrogen recommendations should be tailored to local growing conditions rather than applied uniformly. Multiple studies have suggested that integrating agronomic practices (rather than relying solely upon one) has often proven to be effective in reducing lodging. For example, field studies have demonstrated that combining split nitrogen applications with appropriate plant populations and chlormequat chloride can reduce lodging without significantly decreasing yield; however, responses to these practices have differed among growing seasons. Mangin et al. (2022) reported that the advantage of integrated management was diminished under low rainfall or other environmental stressors, providing further support for the significant effect of growing season weather on management outcomes. There has been a recent shift towards assessing lodging-related traits at the crop-population level rather than at the individual-plant level. Evidence suggests that mixed-cultivar wheat populations generally have exhibited greater lodging stability than monocultures, while maintaining or even increasing grain yield (Kong et al., 2024). Although the underlying physiological mechanisms remain under investigation, complementary differences among cultivars in stem strength, canopy architecture and plant structure are considered likely contributors to this improved stability. Progress has also been made in refining the way plant growth regulators are applied. Recent studies have shown that suitable adjuvants can improve spray performance, allowing effective lodging control with lower amounts of active ingredient. Formulations containing mepiquat chloride or prohexadione-calcium, when combined with compatible adjuvants, have produced satisfactory results at reduced application rates. Also, Matysiak et al. (2025) found that treatment performance differed considerably among years and experimental locations, indicating that environmental conditions continue to influence the consistency of these technologies. Taken together, the available evidence suggests that no single practice provides reliable lodging control across all wheat-growing environments. Durable improvement is more likely to arise from approaches that combine appropriate cultivar selection, balanced nitrogen management and judicious use of growth regulators while accounting for genotype × environment interactions that influence crop performance under field conditions.

## Future research directions

Although decades of research have examined cultivar genetics, crop management practices and environmental conditions, the interactions among these factors that determine yield stability and lodging resistance remain poorly understood. Modern bread wheat cultivars show complex responses to nitrogen availability that cannot be adequately explained by simple additive models. Compared with above-ground stem traits, root anchorage has received less research attention, although it may be critical for preventing lodging under wet soil conditions. There is still insufficient information on root plate development of wheat in response to nitrogen rates and plant populations (Liang et al., 2025). Although recent advances in UAV-enabled high-resolution remote sensing provide opportunities to monitor and scale lodging detection, the practical application of these technologies as decision-support tools remains under development. Machine learning algorithms show promise for grading lodging severity; however, evidence of their reliability across diverse wheat-growing environments remains limited. Machine learning algorithms could be integrated with crop growth models to develop early-warning systems for lodging risk. Given the predicted increase in the frequency and severity of extreme weather events under climate change, further research is needed to better understand genotype × environment × management interactions and their effects on lodging severity in wheat. However, proof-of-concept results from short-term studies on precision nitrogen management, cultivar mixtures and optimized planting dates need to be validated through multi-location and multi-season field evaluations under realistic farming conditions. The response of plant growth regulators to combined environmental stresses is also very uncertain. Although certain classes of chemicals such as Triazoles and quaternary ammonium compounds have been shown to reduce lodging under standard conditions, their performance under shading and nutrient imbalance are still poorly understood. Recent findings indicate that shading suppresses lignin synthesis in wheat, with paclobutrazol only partially compensating, highlighting how much remains to be discovered about regulator performance in complex field environments. Economic and social aspects of lodging management in wheat have likewise received comparatively limited attention. While several agronomic and chemical approaches improve lodging resistance, their profitability under different farm sizes, production costs and market conditions remains insufficiently quantified. More interdisciplinary investigations combining agronomic performance with economic analysis may therefore help develop region-specific lodging-management recommendations that are both practical and economically sustainable. Another challenge lies in the lack of standardized lodging evaluation methods across wheat studies. Existing investigations often use different approaches for lodging measurement, making comparison among studies difficult and limiting the reliability of broader meta-analyses. Development of high-throughput phenotyping systems integrating mechanical measurements, remote sensing and crop phenotyping data may therefore accelerate future breeding and agronomic research focused on improving wheat lodging resistance. Overall, future wheat lodging research will likely require stronger integration of genetics, physiology, agronomy, remote sensing and economic assessment to develop management strategies that are scientifically reliable, environmentally resilient and economically feasible under diverse production conditions.

## Conclusion

Lodging continues to limit yield stability in wheat, particularly under intensive production systems where high nitrogen input, irrigation and vigorous canopy growth increase the likelihood of stem or root failure. Evidence from recent studies indicates that lodging is unlikely to be consistently controlled by a single management approach across contrasting production environments. Canopy structure, stem strength, root anchorage and environmental stress all interact throughout crop development. More consistent lodging management can therefore be achieved by integrating cultivar selection with appropriate agronomic practices and, where suitable, plant growth regulators rather than relying on a single intervention. In wheat, regulators such as uniconazole, trinexapac-ethyl, chlormequat chloride and paclobutrazol have been reported to modify plant architecture and improve lodging resistance, although the magnitude of these responses varies considerably with regulator type, application timing, dosage, cultivar characteristics and prevailing growing conditions (Cigel et al., 2023; Lemanowicz, 2023). UAV platforms, machine-learning classification methods and quantitative lodging indices are increasingly being used to improve the assessment of lodging at field and regional scales, providing information on canopy displacement and affected area that can support more targeted management decisions (Zang et al., 2024). Episodes of strong winds, heavy rainfall and extreme weather events may further increase lodging risk, particularly in high-input wheat systems. Therefore, future lodging management should integrate genetic improvement, balanced agronomic management, targeted use of plant growth regulators and improved phenotyping approaches to maintain yield stability and crop performance under variable environmental conditions.
